# Organometallic Catalysis Catches up with Enzymatic in the Regeneration of NADH

**DOI:** 10.1021/acscatal.5c02162

**Published:** 2025-05-19

**Authors:** Caterina Trotta, Giuseppe Fraschini, Elena Tacchi, Leonardo Tensi, Cristiano Zuccaccia, Gabriel Menendez Rodriguez, Alceo Macchioni

**Affiliations:** a Department of Chemistry, Biology and Biotechnology and CIRCC, 9309 University of Perugia, Via Elce di sotto 8, Perugia 06123, Italy; b Department of Chemical Sciences, University of Padova, Via Marzolo 1, Padova 35131, Italy; c Department of Pharmaceutical Sciences, 9309 University of Perugia, Via del Liceo 1, Perugia 06123, Italy

**Keywords:** NADH regeneration, iridium catalysts, NMR, X-ray diffractometry, organometallic chemistry

## Abstract

A rational design, based on a deep understanding of the reaction mechanism, led to the development of an iridium organometallic catalyst with activity comparable to that of enzymes in the chemical regeneration of NADH, using phosphite as a reducing agent. The innovative structural elements were individuated in replacing pyridine with pyrazine and adding a carbohydrazide moiety in the bidentate amidate supporting ligand. Resulting [Cp*Ir­(pyza-NH_2_)­Cl] (pyza-NH_2_ = κ^2^-pyrazinecarbohydrazide; **1**) outperforms both the analogous complex with pyridine, [Cp*Ir­(pica-NH_2_)­Cl] (pica-NH_2_= κ^2^-pyridincarbohydrazide; **2**), and that missing the −NH_2_ moiety, [Cp*Ir­(pyza)­Cl] (pyza = κ^2^-pyrazineamidate; **3**). A maximum TOF of 13,090 h^–1^ was observed for **1** ([NAD^+^] = 6 mM, [cat] = 7.5 μM, pH 6.58 by phosphite buffer 0.4 M, 313 K), a value never reached for any organometallic catalyst and comparable with that of enzymes. ^1^H diffusion NMR experiments indicate that **1** and **2** undergo dimerization in water through the ionization of the Ir–Cl bond and coordination of the carbohydrazide moiety to a second iridium center. This leads to [Cp*Ir­(pyza-NH_2_)]_2_X_2_ (**1**_**D**_) and [Cp*Ir­(pica-NH_2_)]_2_X_2_ (**2**_**D**_) that were isolated as PF_6_^–^ salts by anion metathesis with NH_4_PF_6_ and fully characterized both in solution (multinuclear multidimensional NMR) and in the solid state (single-crystal X-ray diffractometry). Catalytic NADH regeneration experiments were carried out starting from stock solutions of **1**–**3** complexes in acetonitrile, where diffusion NMR experiments ensure the main presence of mononuclear catalytic precursors, in order to avoid complications due to dimerization. In-depth kinetic studies evidenced that catalyst **1** in combination with the H_2_PO_3_^–^ donor is superior to **2** and **3** in all aspects, facilitating the formation of the Ir–H intermediate and the tendency to donate the hydride to NAD^+^, at the same time inhibiting the detrimental accumulation of the off-cycle cat/NAD^+^ adduct. The introduction of the pyrazine moiety, much less σ-donating and more π-accepting than the pyridine one, is likely responsible for most of the increased activity and stability of **1** with respect to **2**. Meanwhile, the dandling −NH_2_ carbohydrazide moiety might further accelerate the process by providing a basic functionality close to the reactive coordination position and introducing some steric hindrance to hamper the formation of the off-cycle cat/NAD^+^ adduct.

## Graphical abstract

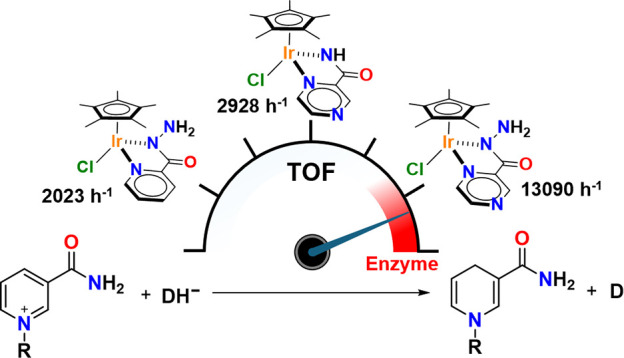

## 1 Introduction

NADH (nicotinamide adenine dinucleotide) is an essential coenzyme ubiquitously present in living cells, acting as a redox carrier of protons and electrons.^1,2^ It plays a pivotal role in any oxidoreductase-mediated biological process and is implicated in regulating the concentration of intracellular reactive oxygen species (ROS). It is also widely employed as a cofactor in important biocatalytic industrial processes.^3–5^ In view of its elevated cost,^6,7^ many strategies have been developed to regenerate NADH from its oxidized form, NAD^+^, based on enzymatic, chemical, electrochemical, and photochemical processes.^8–13^ As for the chemical regeneration of NADH, organometallic catalysts have been successfully employed in combination with inexpensive reducing agents such as formate (HCOO^–^) or phosphite (H_2_PO_3_^–^), acting as hydride donors (DH^–^, eq 1).^14–19^

$${\mathrm{NAD}}^{+}+{\mathrm{DH}}^{-}\to \mathrm{NADH}+D$$ 1

The utilization of organometallic catalysts offers considerable advantages, including the possibility of tuning their activity, selectivity, and stability through a suitable choice of the metal and rational design of the first and second coordination sphere.^20^ Indeed, last decades have witnessed a tremendous improvement of performances of organometallic catalysts for the chemical regeneration of NADH.^21–24^ Focusing on organoiridium catalysts, TOF increased from 54 h^–1^ of [Cp*Ir^III^(4-(1*H*-pyrazol-1-yl-κN^2^)­benzoic acid)­(H_2_O)]­SO_4_ reported by Fukuzumi et al. in 2012 ^14^ to a maximum of 7825 h^–1^, reported for [Cp*Ir^III^(pba)­Cl] (pba = 4-(picolinamido)­benzoic acid) by Qi et al. in 2021 (Figure 1).^16^

**Figure 1:**
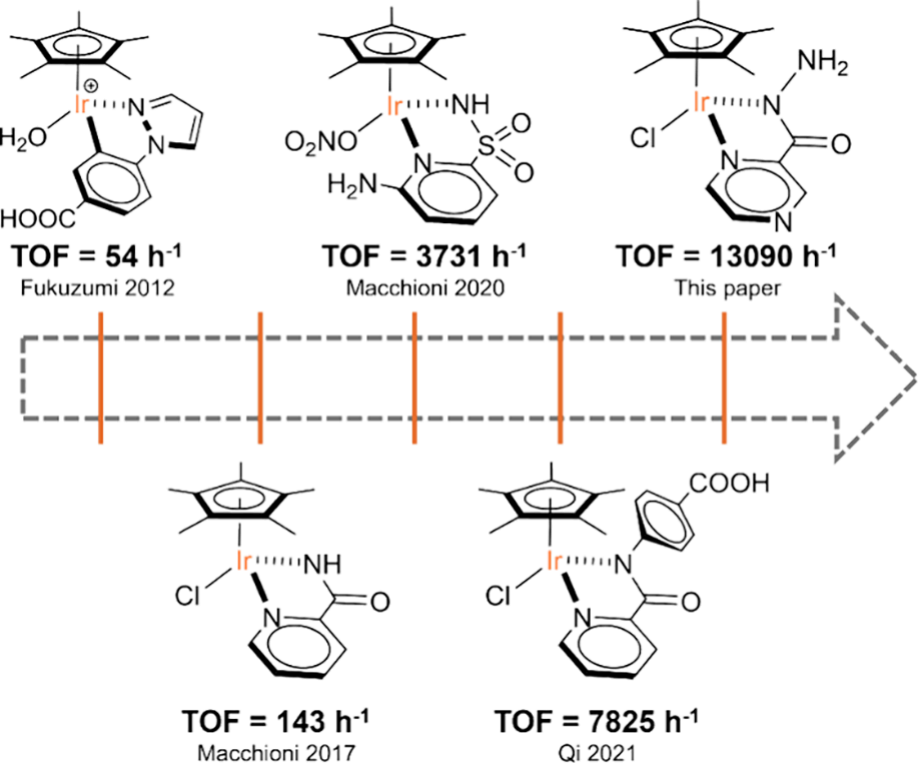
Most efficient Ir catalysts for NADH regeneration.

Our group contributed to this pathway developing Cp*Ir^III^ catalysts bearing N,N or N,O bidentate ligands through a rational approach driven by the understanding of the effect of electronic and structural factors on performance, in terms of TOF, selectivity toward 1,4-NADH and TON.^17–19^ Three main factors were individuated: (i) the utilization of an electron-withdrawing ligand to enhance the metal acidity; (ii) the incorporation of a basic, and (iii) hindering group in proximity to the catalytic site. Indeed, in 2020, we showed that the substitution of a pyridine-amidate bidentate ligand with the less electron-donating pyridine-sulfonamidate one, thus moving from [Cp*Ir­(R-pica)­NO_3_] (pica = κ^2^-pyridine-2-amidate = picolinamidate) to [Cp*Ir­(R-pysa)­NO_3_] (pysa = κ^2^-pyridine-2-sulfonamidate), led to a substantial improvement of the catalytic activity. Furthermore, the introduction of a −NH_2_ functionality in the 6-position of the pyridine ring, [Cp*Ir­(6-NH_2_-pysa)­NO_3_], was found to be a crucial factor, resulting in a record TOF of 3731 h^–1^ (313 K) (Figure 1), at that time.^18^

Extensive kinetic studies combined with theoretical investigations enabled us to propose a reaction mechanism that is coherent with all experimental observations. It comprises two central chemical steps (Figure 2). First, a metal hydride intermediate (**Ir_H**) is formed from the reaction of **Ir_H**_**2**_ **O**, the starting form of the catalyst, with the hydride donor. This is generally accounted as the turnover limiting step of the reaction. It is followed by hydride transfer from **Ir_H** to NAD^+^, leading to the release of 1,4-NADH and regeneration of **Ir_H**_**2**_ **O**. The latter might associate with NAD^+^, through hydrogen bonding, forming an out-of-cycle species (**Ir_NAD** ^**+**^), which is detrimental since it subtracts catalytic centers (Figure 2).^17^

**Figure 2:**
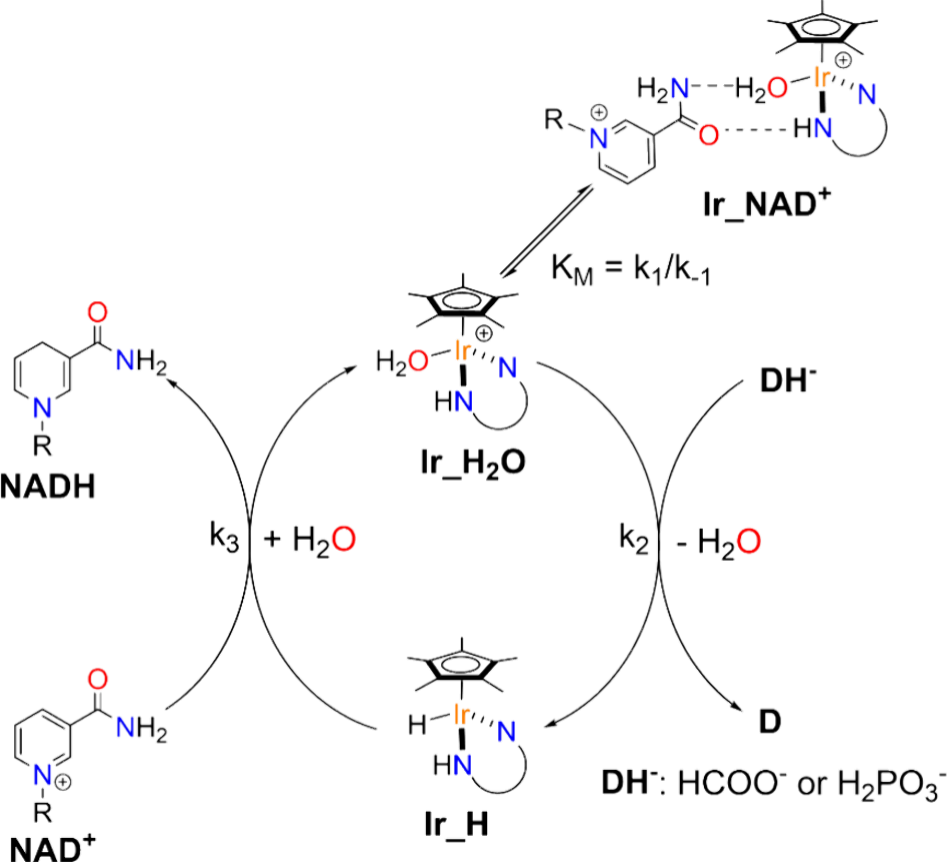
Reaction mechanism proposed for the regeneration of NADH mediated by [Cp*Ir^III^(N,N)­X] catalysts.

The mechanism illustrated in Figure 2 rationalizes the factors described before, in that the presence of less donating bidentate ligands increases the acidity of the metal center and, consequently, facilitates the C–H or P–H activation step leading to **Ir_H**. The introduction of a basic functionality in proximity of the reactive coordination position might act as a shuttle of the hydride to the 4-position of NADH and, at the same time, inhibit the formation of **Ir_NAD** ^**+**^. While mathematical equations derived from the proposed reaction mechanism of Figure 2 adequately fit experimental kinetic data, providing reasonable *k*_1_–*k*_3_ values, they also suggest the presence of an additional species between **Ir_H**_**2**_ **O** and **Ir_H** for pyrdine-sulfonamidate catalysts. In-depth kinetic studies suggest that such species derive from the dissociation of the pyridine ring from the metal center that makes the catalytic system more fragile, possibly limiting its duration.^19^

With the aim of adjusting the i–iii factors for having high TOF without affecting the stability of the catalytic system, thus avoiding limitations in terms of TON, we focused our attention on introducing the pyrazineamidate (pyza) bidentate ligand in the first coordination sphere of iridium. The rationale behind such a choice stems from the peculiar properties of pyrazine, which is much less σ-donating (p*K*_a_ of pyrazinium and pyridinium are 0.6 and 5.23, respectively)^25^ and more π-accepting than pyridine.^26,27^ This should lead to an increased acidity of the metal center without weakening the Ir–N bond due to its partial double-bond character derived from the π-back-donation. Furthermore, the carbohydrazide moiety directly provides a basic functionality close to the reactive coordination position and some steric hindrance to hamper the formation of the off-cycle adduct.

Herein, we report the synthesis, characterization, and application in the catalytic regeneration of NADH of [Cp*Ir­(pyza-NH_2_)­Cl] (pyza-NH_2_ = κ^2^-pyrazinecarbohydrazide; **1**) along with two related complexes: [Cp*Ir­(pica-NH_2_)­Cl] (pica-NH_2_ = κ^2^-pyridinecarbohydrazide; **2**), featuring pyridine in place of pyrazine, and [Cp*Ir­(pyza)­Cl] (pyza = κ^2^-pyrazineamidate; **3**), which lacks the −NH_2_ group (Figure 3). Contrasting the activity of **1**–**2** and **1**–**3** sheds light on the effect of the two structural modifications and reveals possible synergism between them.

**Figure 3:**
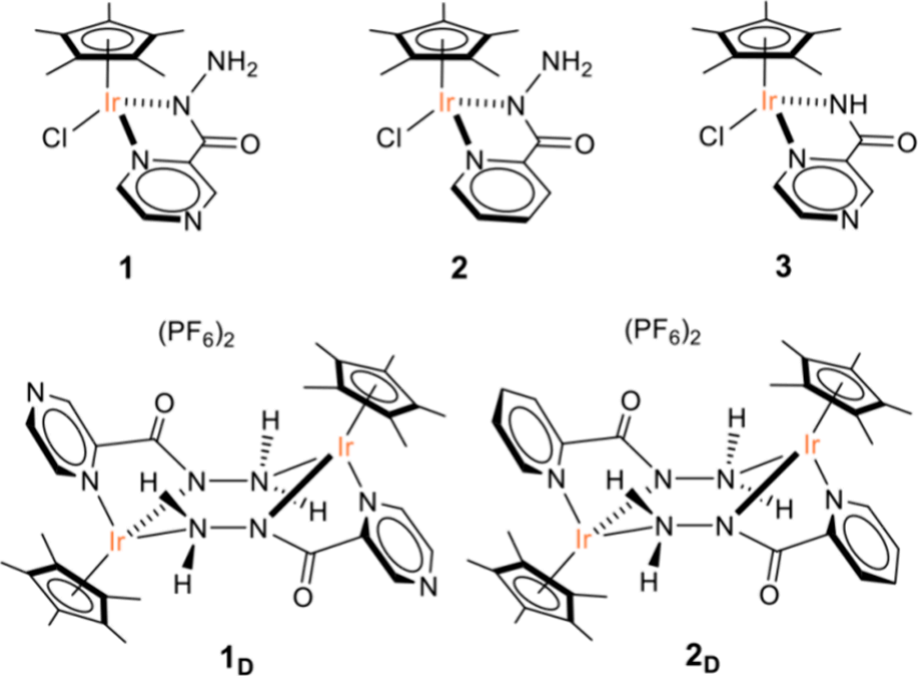
Sketch of the chemical structures of the complexes investigated.

## 2 Results

### 2.1 Synthesis and NMR Characterization

Pyza-NH_2_ and pica-NH_2_ ligands were prepared according to the literature.^28,29^ Complexes **1** and **2** were synthesized in methanol by the reaction of the precursor [Cp*IrCl_2_]_2_ with 2 equivalents of the suitable ligand, in presence of an equal molar amount of KOH (Figure 4 and Supporting Information (SI)). Complex **3** was synthesized as reported elsewhere.^30^

**Figure 4:**
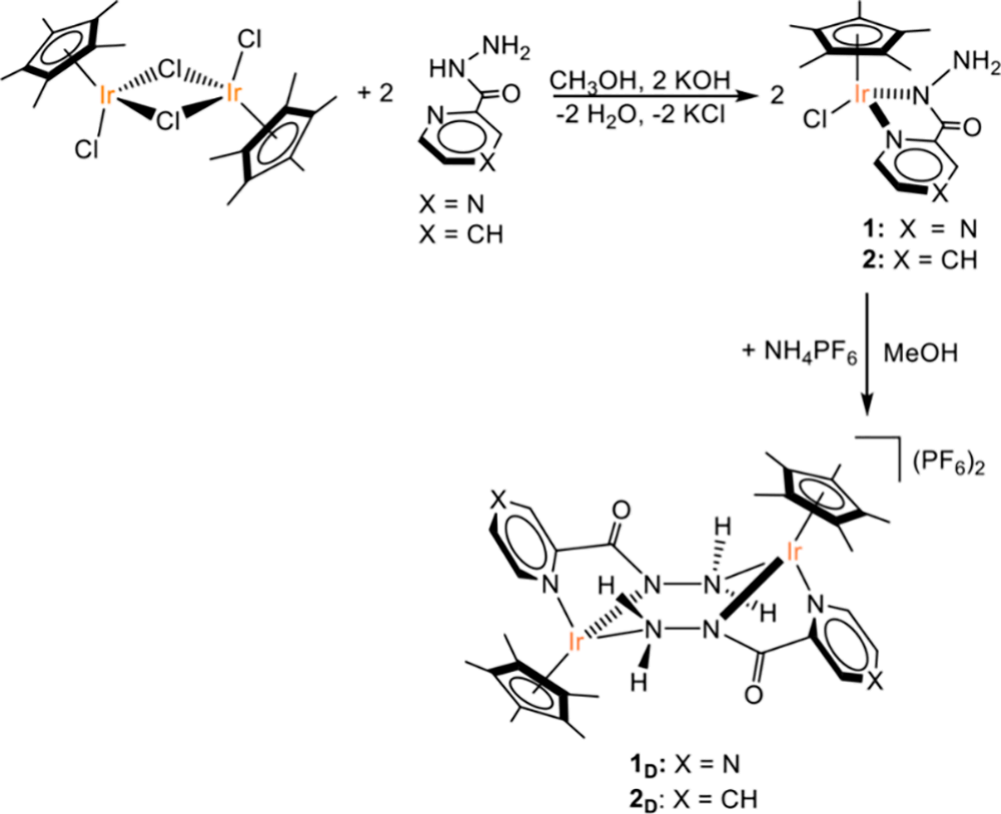
Synthetic procedure for **1**, **2** and **1**_**D**_, **2**_**D**_.

Complexes **1**–**3** were characterized in solution by 1D and 2D multinuclear NMR spectroscopy in DMSO-d_6_ (Table 1 and the SI). The methodology for resonance assignment, detailed in the following for **1**, has been applied to all other complexes. The ^1^H NMR spectrum of **1** (Figure S4) revealed the presence of a single set of resonances with the most shielded one being a singlet at δ_H_ = 1.71 ppm, which was assigned to the methyl groups of the Cp* ligand by integration and typical chemical shift (H_10_).^31–34^ The aromatic region of the spectrum shows three typical resonances of the pyrazine ring. One of these showed a strong NOE with H_10_ and was assigned to H_6_ (Figure 5a). H_5_ was identified owing to a strong dipolar interaction with H_6_ (Figure 5b). The remaining aromatic proton was, consequently, assigned to H_3_. A broad signal, integrating for two protons, was observed at δ_H_ = 5.17 ppm and assigned to the NH_2_ moiety (H_8_) (Figure S4). Carbon resonances were assigned owing to their scalar correlations with protons detected in the ^1^H,^13^C HSQC NMR spectrum (Figure 5c). Interestingly, the proton in the *ortho* position of the pyrazine ring (H_6_) is not the most up-shifted resonance, as it is often observed in pyridine ring systems (Figure S4).

**Figure 5:**
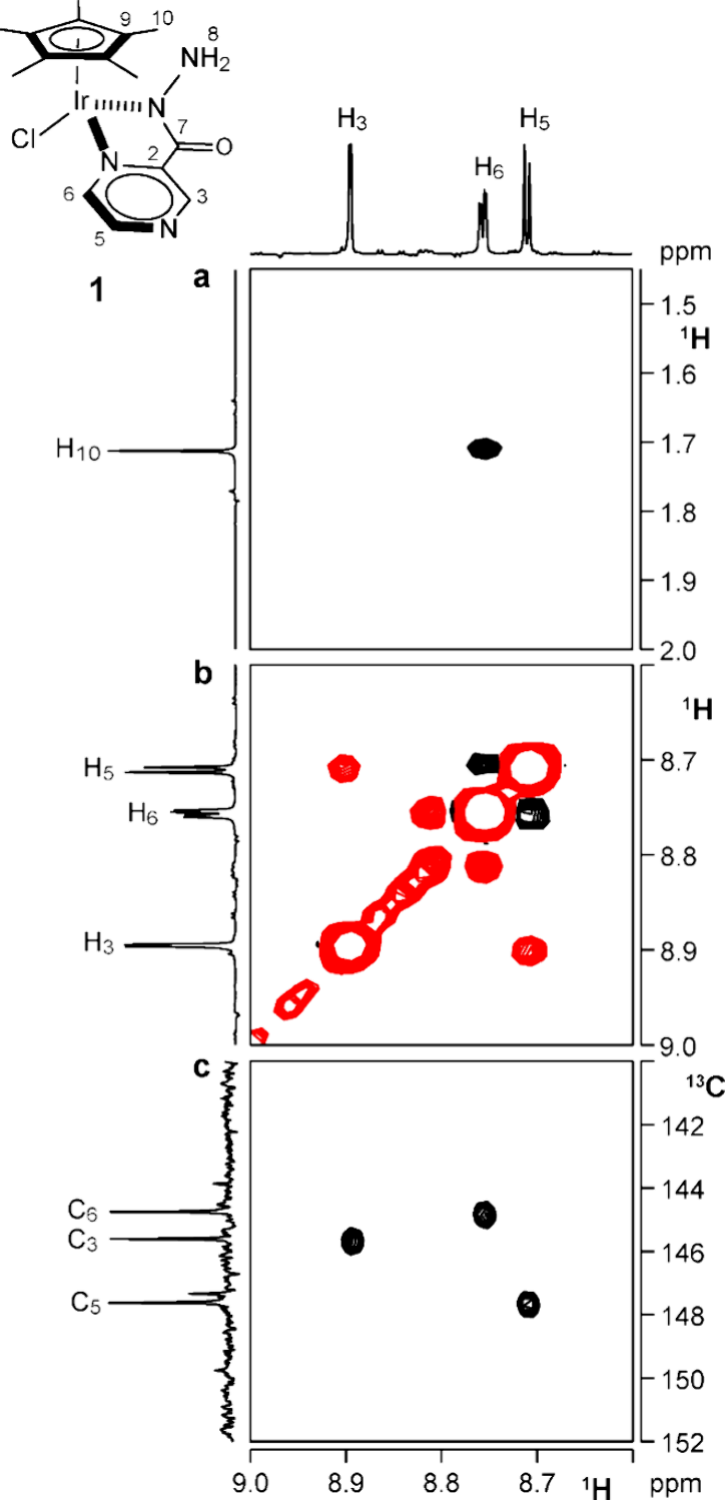
(a) Section of the ^1^H NOESY NMR spectrum showing a NOE contact between H_6_ and H_10_ protons (DMSO-d_6_, 298 K). (b) Section of the ^1^H NOESY NMR spectrum of **1** showing NOE contacts between the aromatic protons H_5_ and H_6_ (DMSO-d_6_, 298 K). (c) Section of the ^1^H,^13^C HSQC spectrum of **1** showing the direct scalar correlations of aromatic protons and carbons (DMSO-d_6_, 298 K).

**Table 1:** ^1^H and ^13^C Chemical Shifts of the Investigated Complexes and Δ Chemical Shifts (ppm) between **1**–**2** and **1**_**D**_–**2**_**D**_

	δ_H_	δ_H_	δ_H_	Δδ_H_	δ_C_	δ_C_	Δδ_C_
	DMSO-d_6_	DMSO-d_6_	D_2_O	DMSO-d_6_	DMSO-d_6_	DMSO-d_6_	DMSO-d_6_
	**1**	**1**_**D**_	**1**_**D**_	**1/1**_**D**_	**1**	**1**_**D**_	**1/1**_**D**_
2					147.5	147.5	0
3	8.89	9.09	8.55	0.20	145.6	149.8	4.2
4							
5	8.71	8.89	8.92	0.18	147.6	146.4	1.2
6	8.75	8.80	9.17	0.05	144.8	146.1	1.3
7					162.4	164.4	2.0
8	5.17	5.38		0.21			
9					88.2	96.6	8.4
10	1.71	1.76	1.75	0.05	8.9	8.5	0.4
	**2**	**2**_**D**_	**2**_**D**_	**2/2**_**D**_	**2**	**2**_**D**_	**2/2**_**D**_
2					153.6		
3	7.74	7.97	7.64	0.23	123.8	126.0	2.2
4	8.03	8.29	8.02	0.26	139.5	142.0	2.5
5	7.55	7.76	7.28	0.21	126.8	129.2	2.4
6	8.65	8.72	8.91	0.07	151.3	152.8	1.5
7					163.9	165.8	1.9
8	5.14	5.28		0.14			
9					87.1	95.9	8.8
10	1.69	1.76	1.76	0.07	9.0	8.5	0.5

As catalytic experiments were carried out in water, additional NMR studies in this solvent were performed. The ^1^H NMR spectra of **1** in D_2_O show a single set of resonances with a remarkable shift of some hardly attributable to a solvent effect (Table 1). Indeed, diffusion NMR spectroscopy (*vide infra*) strongly suggests that **1** transforms into a dinuclear species (**1**_**D**_) (Table 1 and Figure 8). The same occurs for **2** that generates **2**_**D**_, in water. **1**_**D**_ and **2**_**D**_ were isolated by the reaction of **1** and **2**, respectively, in methanol with an excess of NH_4_PF_6_ (SI) (Figure 4) and characterized in DMSO-d_6_. The assignment of the resonances of **1**_**D**_ and **2**_**D**_ follows the same reasoning than that illustrated above for **1**. The ^1^H resonances exhibiting the most significant shift passing from monomeric to dimeric species are H_5_ (Δ = 0.18 ppm), H_3_ (Δ = 0.20 ppm), and H_8_ (Δ = 0.21 ppm) for **1**/**1**_**D**_ and H_5_ (Δ = 0.21 ppm), H_4_ (Δ = 0.26 ppm), and H_3_ (Δ = 0.23 ppm) for **2**/**2**_**D**_ (Table 1). In the ^13^C NMR spectra, relevant shifts were observed for C_9_ (Δ = 8.4 and 8.8 ppm) and C_3_ (Δ = 4.2 and 2.2 ppm) (for **1/1**_**D**_ and **2/2**_**D**_, respectively) but also C_5_ (Δ = 2.4 ppm) in the case of **2**/**2**_**D**_ (Table 1). Higher chemical shift values observed for quaternary C_9_ carbon of Cp* in dinuclear species might indicate weaker Cp*–Ir bonding and are consistent with a lower degree of shielding of aromatic protons near Cp*, which, indeed, fall at higher chemical shifts with respect to those in the monomeric species. The significant differences in chemical shifts between **1**_**D**_ in DMSO-d_6_ and D_2_O, particularly for H_6_ and H_3_ (Table 1), are difficult to attribute to solvent change and might be due to the presence of ion pairing or supramolecular cationic aggregation in the former.^35^

Interestingly, adding 250 mM KCl to 5 mM solutions of **1**_**D**_ and **2**_**D**_ in D_2_O caused the instantaneous formation of **1** and **2** in 58:42 and 79:21 ratios, respectively (Figure 6). The equilibrium constants (*K*_eq_) were estimated by integration of the resonances of the two species to be 7.4 for **1**/**1**_**D**_ and 282 for **2**/**2**_**D**_, which corresponds to an exergonic Δ*G* ^0^ = −1.19 and −3.52 kcal/mol, respectively, for the dimerization process.

**Figure 6:**
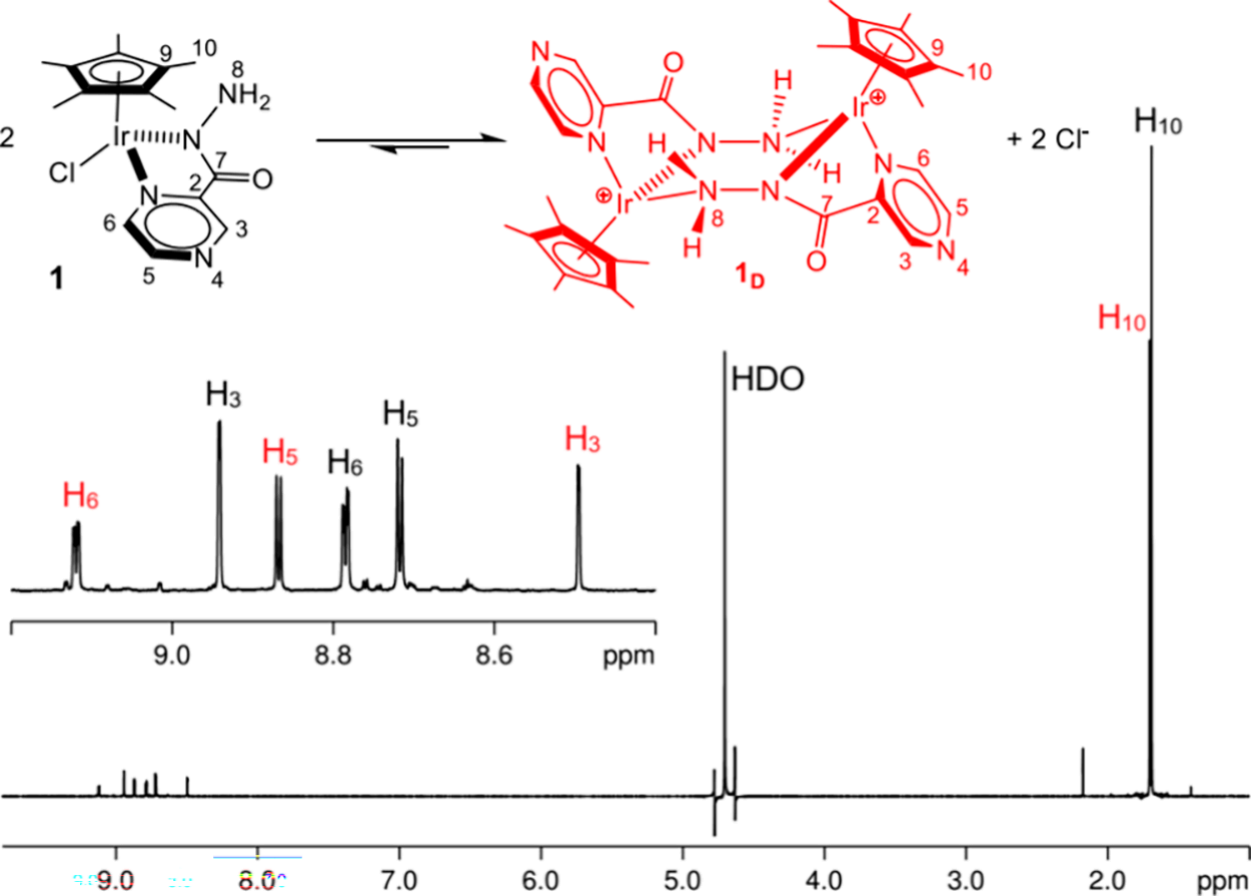
Dimerization reaction of complex **1** leading to **1**_**D**_ (top); ^1^H NMR spectrum of a mixture of **1** and **1**_**D**_ in D_2_O at 298 K.

Exchange cross peaks were observed in the ^1^H EXSY NMR spectrum of those solution (Figure 7), indicating that monomeric and dinuclear species reversibly interconvert into each other in the NMR time scale. Their quantification allowed calculation of the rate constant for the dimerization process: *k*_exsy_ = 0.02 s^–1^ for **1**/**1**_**D**_ and 0.2 s^–1^ for **2**/**2**_**D**_.

**Figure 7:**
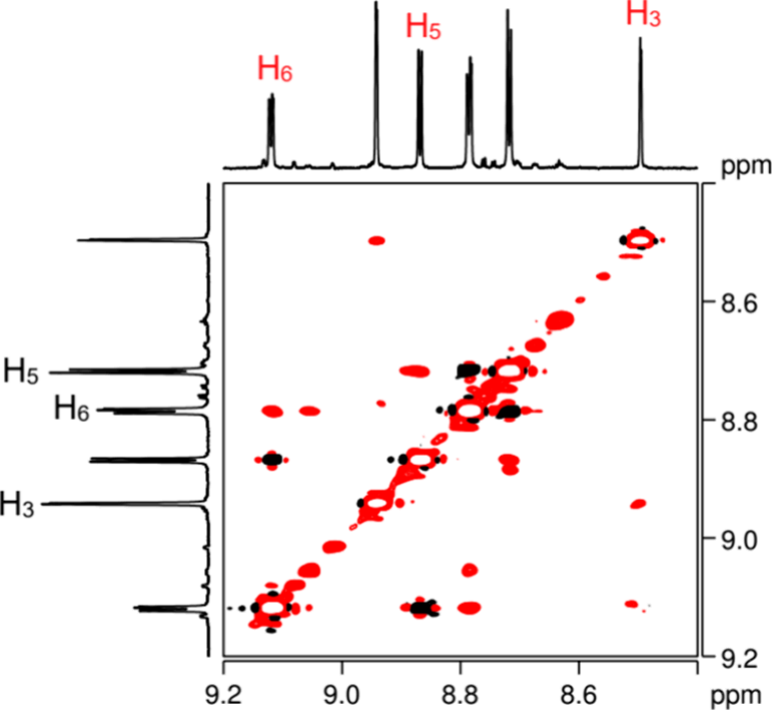
A section of the ^1^H,^1^H EXSY NMR spectrum (D_2_O, 298 K) showing the exchange cross peaks between resonances of **1** (black) and those of **1**_**D**_ (red).

### 2.2 Diffusion NMR Spectroscopy

Pulse gradient spin echo (PGSE) NMR experiments were performed to assess the level of aggregation of the complexes in solution. Studies were carried out in DMSO-d_6_, the solvent of main characterization, CD_3_CN used for preparing stock solutions to inject in catalytic experiments, and D_2_O, the reaction medium of catalysis. PGSE NMR experiments allowed the translational self-diffusion coefficient (*D*_t_) to be measured, from which hydrodynamic volume (*V*_H_) was derived, passing through a modified version of the Stokes–Einstein equation.^36–40^ Data are reported in Table 2.

**Table 2:** Translation Self-Diffusion Coefficients and Hydrodynamic Volumes of **1**, **1**_**D**_, **2**, and **2**_**D**_ Measured by PGSE NMR in Various Solvents

entry	catalyst	solvent	*D*_t_ (×10^–10^ m^2^ s^–1^)	*V*_H_ (Å^3^)
**1**	**1**_**D**_ ^a^	D_2_O	3.54	864 ± 130
**2**	**2**_**D**_ ^a^	D_2_O	3.50	847 ± 127
**3**	**1** ^b^	D_2_O	4.42	505 ± 76
4	**1**_**D**_	DMSO-d_6_	0.17	1052 ± 158
**5**	**2**_**D**_	DMSO-d_6_	0.20	767 ± 106
6	**1**	CD_3_CN	1.45	572 ± 86
7	**2**	CD_3_CN	1.41	613 ± 92
8	**1**_**D**_	CD_3_CN	1.06	1232 ± 185
9	**2**_**D**_	CD_3_CN	1.12	1098 ± 165

a Obtained upon dissolution of **1** and **2** in D_2_O.

b Obtained upon addition of KCl to a solution of **1**_**D**_ in D_2_O.

As anticipated, diffusion NMR experiments carried out for **1** and **2** complexes in D_2_O led to determine a *V*_H_ close to 850 Å^3^ (Table 2, entries 1 and 2), much higher than that expected for a mononuclear species,^41–43^ explainable with the presence of dinuclear **1**_**D**_ and **2**_**D**_. It is known that *V*_H_ is usually 1.3–1.4 times the volume of Van der Waals (*V*_VdW_) and somewhat smaller than the crystallographic volume (*V*_XRay_), both deducible from the X-ray structures of compounds. *V*_VdW_ amounts to 729.3 and 741.2 Å^3^ for **1**_**D**_ and **2**_**D**_, respectively, whereas *V*_XRay_ are 947.5 and 967.7 Å^3^. Since in polar solvents, especially if protic, the counterions are reasonably not paired with the cation, it is useful to consider that *V*_VdW_ of the cationic moieties of **1**_**D**_ and **2**_**D**_ amount to 585.9 and 597.5 Å^3^, respectively. Consequently, determined *V*_H_ volumes around 850 Å^3^ nicely fit with having the predominant presence in D_2_O of unpaired cationic dinuclear species.

In DMSO-d_6_ (Table 2, entry 4) and CD_3_CN (Table 2, entries 8 and 9), measured *V*_H_ is significantly higher than that expected for a cationic dinuclear species. ^19^F PGSE NMR experiments provide a *V*_H_(PF_6_^–^) = 106 Å^3^ consistent with the presence of solvated counterion not paired with the cation. This indicates that the observed increase in *V*_H_ is not due to ion pairing of the cationic dinuclear species with PF_6_^–^ but rather to some self-aggregation of the dinuclear species, which is more marked for **1**_**D**_ than **2**_**D**_. The latter appears to be not aggregated at all in DMSO-d_6_, exhibiting a *V*_H_ even smaller than that in D_2_O (Table 2, entry 5).

Finally, *V*_H_ for monomeric species **1** (Table 2, entries 3 and 6) and **2** (Table 2, entry 7) in CD_3_CN are consistent with what is expected from the solid-state structure of **2**. Indeed, *V*_VdW_ and *V*_XRay_ are equal to 323.4 and 421.3 Å^3^, respectively, which well compare to the observed *V*_H_, even if some self-aggregation appears to be present in **1**_**D**_ and **2**_**D**_ (observed *V*_H_ are in the 500–600 Å^3^) (Figure 8).

**Figure 8:**
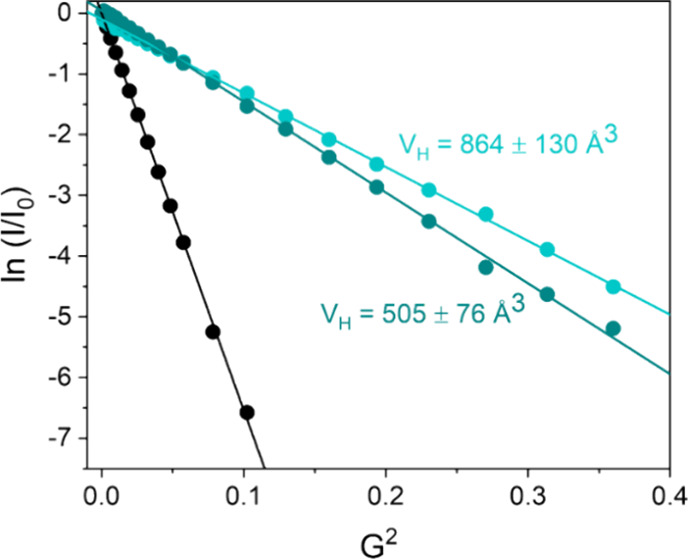
Semilogarithmic plot of ln­(*I*/*I*_0_) versus *G* ^2^ for **1** (green) and **1**_**D**_ (light green) in D_2_O.

### 2.3 X-Ray Single-Crystal Solid-State Characterization

Single crystals of complexes **1**_**D**_, **2**_D_, and **2** of good quality for X-ray diffraction studies were obtained through slow diffusion of diethyl ether in methanolic solutions. The solid-state structures of **1**_**D**_ and **2**_**D**_ are shown in Figure 9 while the structure of **2** is reported in Figure S20. Despite our repeated efforts, we were unable to obtain single crystals of **1** that were suitable for X-ray analysis.

**Figure 9:**
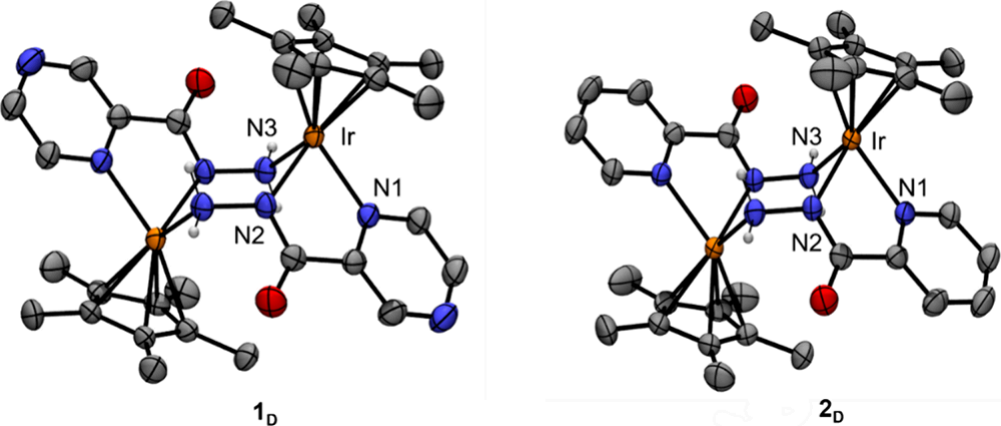
ORTEP drawing of cations **1**_**D**_ and **2**_**D**_. Ellipsoid at 50% probability, hydrogen atoms, and counterions are omitted for clarity. Color code: Ir = orange, *N* = blue, O = red, C = gray.

**1**_**D**_ and **2**_**D**_ crystallized as bicationic bimetallic complexes with a piano stool geometry at iridium, which is coordinated by the Cp* ligand and pyza or pica bidentate ligand, whereas the hydrazide dangling groups act as bridging neutral ligands. Relevant bond lengths and angles for **1**_**D**_, **2**_**D**_, and **2** and those of previously measured for **3** ^30^ are reported in Table 3.

**Table 3:** Relevant Bond Lengths (Å) and Bond Angles (°) for **1**_**D**_, **2**, **2**_D_, and **3**

	1_D_	2	2_D_	3
Ir–Cp*	1.802	1.770	1.797	1.794
**Ir–N1**	2.109	2.110	2.119	2.085
**Ir–N2**	2.085	2.057	2.081	2.071
**Ir–N3**	2.156		2.147	
**N2–N3**	1.435		1.440	
**Ir–Cl**		2.402		2.411
Cp*–Ir–N1	126.16	136.18	126.14	133.74
Cp*–Ir–N2	132.17	136.74	131.25	132.10
Cp*–Ir–N3	131.21		131.42	
Cp*–Ir–Cl		119.87		125.53
**N1–Ir–N2**	75.34	75.75	75.23	76.22
**N2–Ir–N3**	81.92		82.87	
**Ir–N2–N3**	127.69	124.12	127.16	
**Ir–N3–N2**	113.02		122.36	

The Cp* and the N,N ligands are disposed in a pseudo-*trans* configuration with respect to the plane of the Ir-N2-N3-Ir′-N2′-N3′ ring while the two bridging ligands are arranged to form a six-membered ring, similar to the structure of a chair-conformed cyclohexane, with Ir-N2-N3 angles of 127.69 and 127.16° and Ir-N3′-N2’ angles of and 113.02 and 112.36°, for **1**_**D**_ and **2**_**D**_, respectively. Although the crystal structures of complexes having six-membered rings of the M-N-N-M type are already known for Cd,^44,45^ Al,^46^ Ru,^47^ Fe,^48^ and Ni^49^ complexes, this is, to the best of our knowledge, the first example of a six-membered ring consisting of two iridium and four nitrogens. Ir-Cp* centroid distances in **1**_**D**_ (1.802 Å) and **2**_**D**_ (1.797 Å) are significantly longer than those in **2** (1.770 Å) and **3** (1.784 Å) (Table 3) in agreement with the consideration done in the NMR section. Notably, the Ir-N1 bond in **1**_**D**_ (2.109 Å) is relevantly shorter than that of **2**_**D**_ (2.119 Å), consistently with the back bonding of the metal over the pyrazine ring. For both compounds, the Ir-N2 bond (2.085 Å for **1**_**D**_ and 2.081 Å for **2**_**D**_) is found to be shorter than the bridging Ir-N3 bond (2.156 Å for **1**_**D**_ and 2.147 Å for **2**_**D**_) in agreement with the weaker donating abilities of the NH_2_ moiety with respect to the X-type bonding of the amidic nitrogen. Bond lengths analogous to those presented in this study have been reported for iridium and rhodium bimetallic complexes with a single tetradentate picolinic hydrazide ligand ([(Cp*MCl)_2_ κ^4^N,NμN,O]­PF_6_ with M = Ir, Rh) (2.083 Å and 2.127 Å for the Ir-N2 and Ir-N3 bond, respectively).^50^ The crystal structure of **2** (Figure S20) showed that the monomeric complex is arranged in a piano stool conformation with a chloride and the pyridine carbohydrazide ligand in the first coordination sphere. It is noteworthy that the Ir-N1 bond length of **2** (2.110 Å) is somewhat shorter than that of **2**_**D**_, due to the presence of the more electron withdrawing chloride anion coordinated to the metal center.

### 2.4 Catalytic Activity

#### UV–Vis Experiments

Complexes **1**–**3** were tested as catalysts for NADH regeneration (equation 1) at 313 K, using phosphite and formate as sources of hydride, in a water solution at pH close to neutrality by phosphite and phosphate buffer, respectively. The course of the reaction was monitored by following the appearance of the diagnostic absorption band of 1,4-NADH at 340 nm (ε = 6220 M^–1^ cm^–1^).

In a typical experiment, the concentration of NADH was 4 mM and that of the catalyst was 7.5 μM, whereas the strength of the buffer was 0.4 M (phosphite) and 0.1 M (phosphate) (Figure 10). Catalysis was initiated by the addition of complexes **1**–**3** dissolved in acetonitrile, where PGSE NMR studies indicated the main presence of monomeric species even at the concentration of the stock solutions (2–3 mM). The decision of using monomeric precursors was derived from comparative catalytic tests, performed under identical conditions, starting from **1** and **1**_**D**_, using both HCOO^–^ and H_2_PO_3_^–^ as hydride donors. Similar catalytic activity was observed with HCOO^–^, whereas the activity was much higher with **1** than with **1**_**D**_ when H_2_PO_3_^–^ was employed (Figure 11, Figure S21, and Tables S2 and S3).

**Figure 10:**
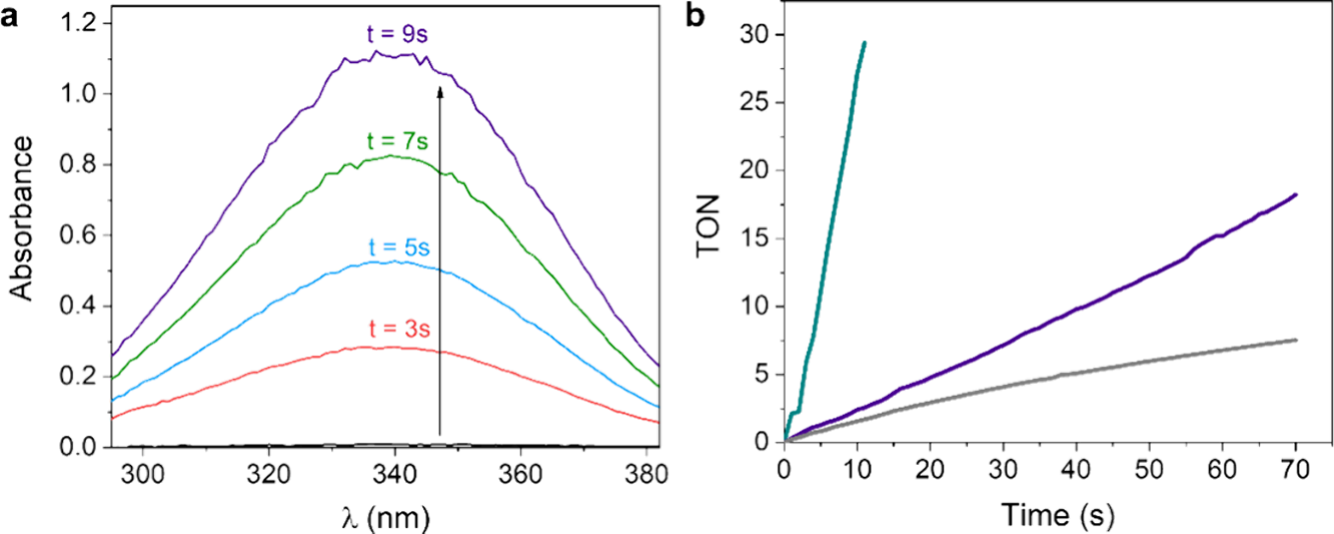
(a) Kinetics of the growth of the absorption band of NADH at 340 nm. (b) TON vs *t* course obtained by means of UV–vis spectroscopy for **1** (green), **2** (gray), and **3** (purple) at [cat] = 7.5 μM, [NAD^+^] = 4 mM; phosphite buffer 0.4 M pH 6.58, 313 K.

**Figure 11:**
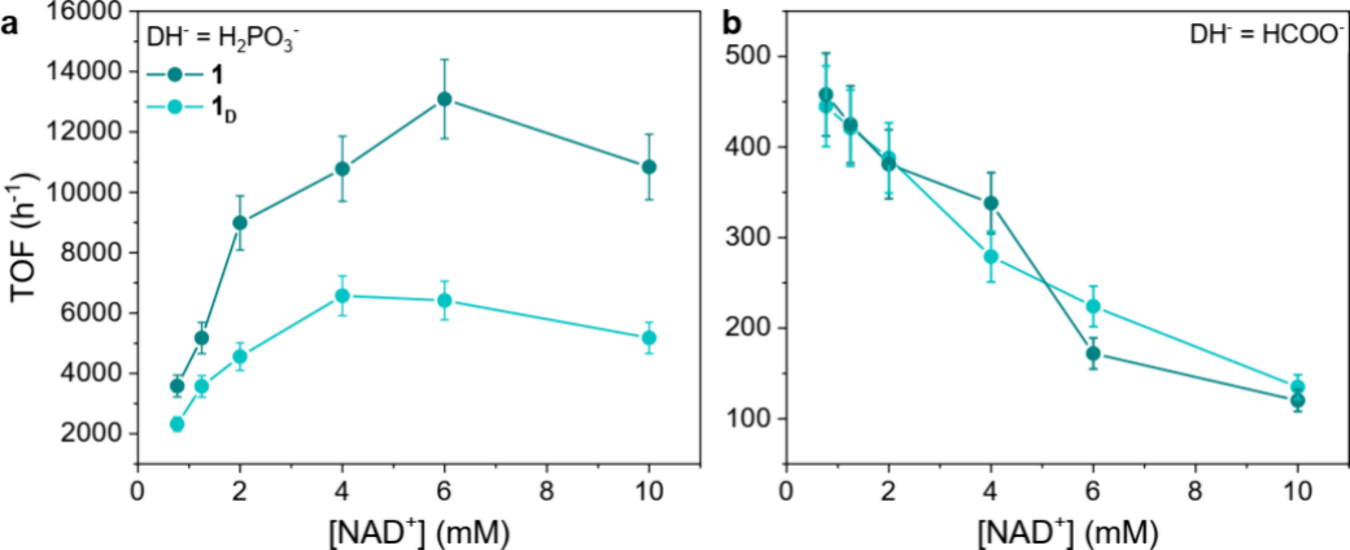
(a) TOF vs [NAD^+^] plot for catalysts **1** and **1**_**D**_ ([cat] = 7.5 μM, phosphite buffer 0.4 M pH 6.58, 313 K). (b) TOF vs [NAD^+^] plot for catalysts **1** and **1**_**D**_ ([cat] = 7.5 μM; [HCOOK] = 0.125 M; phosphate buffer pH 7 0.1 M, 313 K).

The reasons of these observations might be found in the higher aptitude of HCOO^–^ to break the dimers or to the fact that the turnover limiting step for HCOO^–^ is C–H activation, whereas for H_2_PO_3_^–^ it is the hydride transfer to NAD^+^, at least at a low NAD^+^ concentration. In any case, using monomeric precursors in CH_3_CN avoids the complication of taking into account dimer dissociation for generating the active species. Once introduced in the reactor, the catalyst concentration is so low (typically 7.5 μM) that dimer formation appears to be unlikely also in water. This means that for the discussion of catalytic results, acquired under the conditions illustrated above, dimer formation can be safely neglected.

Catalytic experiments were performed using **1**–**3** varying NAD^+^ concentrations (0.25–10 mM), catalyst concentrations (2.5–10 μM), and nature of hydride donor at pH close to neutrality and 313 K (SI). All catalytic experiments performed by changing the concentration of catalysts exhibited a first order on the catalyst, meaning that TOF remained substantially the same when the concentration of complexes was varied (Figures S22–S24).

As for the activity as a function of NAD^+^ concentration, a completely different trend was observed depending on the nature of the H-donor. When H_2_PO_3_^–^ is used, an initial increase of TOF was observed enhancing the NAD^+^ concentration followed by a decrease when the latter reached 0.25 mM for **2**, 0.5 mM for **3**, and 6 mM for **1** (Figure 12a). For the latter at [NAD^+^] = 6 mM, a record TOF = 13,090 h^–1^ was observed, much higher than the TOF max observed for **2** (TOF = 2023 h^–1^, [NAD^+^] = 0.25 mM), in which pyridine is present instead of pyrazine, and **3** (TOF = 2928 h^–1^, [NAD^+^] = 0.5 mM), having the pyrazine ring but missing the −N–NH_2_ moiety (Figure 12a). These results clearly evidence that both structural motives, pyrazine instead of pyridine and the −N–NH_2_ moiety, must be contemporary present in order to maximize performance. TOF in the case of HCOO^–^ as the H-donor is considerably smaller than with H_2_PO_3_^–^, especially at [NAD^+^] > 0.5 mM, and it decreases by increasing the NAD^+^ concentration (Figure 12 b). The activity order is also different: **2** ≈ **3** > **1**.

**Figure 12:**
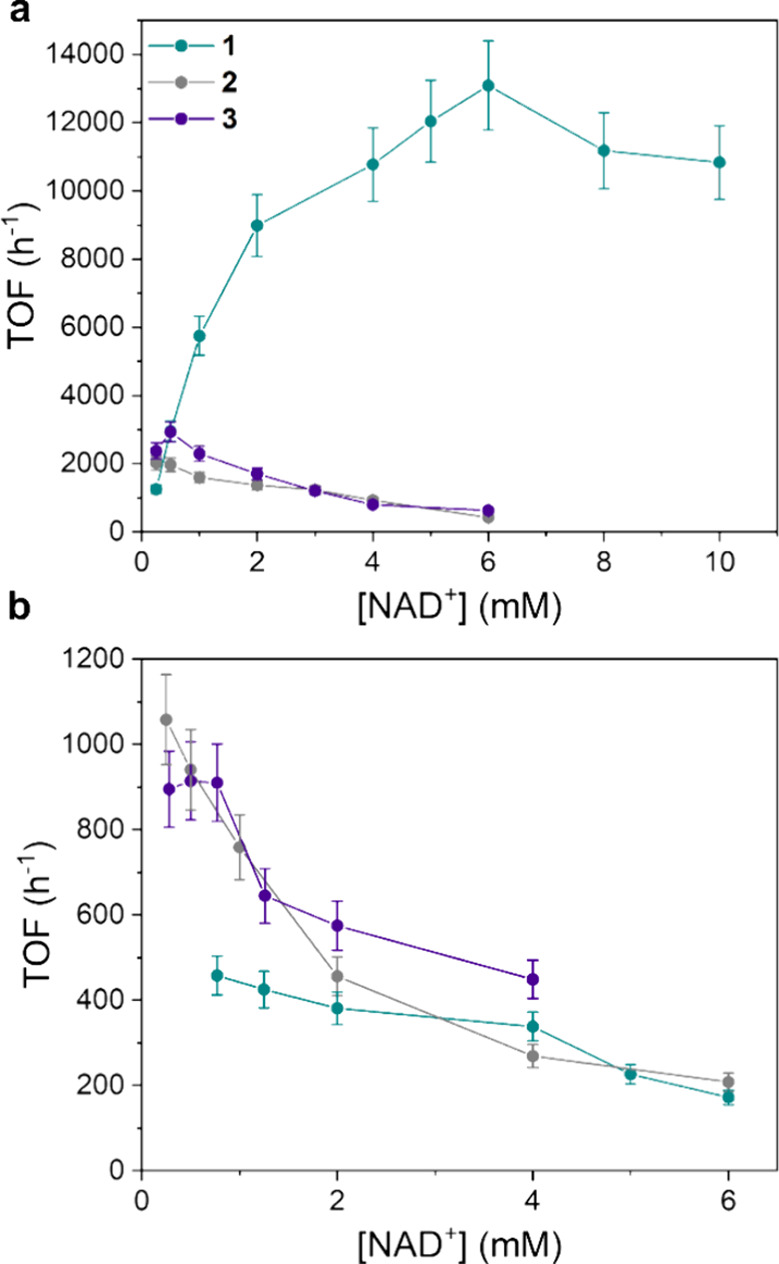
(a) TOF vs [NAD^+^] plot for catalysts **1**–**3** ([cat] = 7.5 μM, phosphite buffer 0.4 M, pH 6.58, 313 K). (b) TOF vs [NAD^+^] plot for catalysts **1**–**3** ([cat] = 7.5 μM; [HCOOK] = 0.125 M; phosphate buffer pH 7 0.1 M, 313 K).

The stability of best-performing catalyst **1** was explored by carrying on an experiment with almost 100 times lower catalyst concentration (90 nM) ([NAD^+^] = 2.0 mM, 313 K, phosphite buffer 0.4 M, pH 6.58). **1** maintains all its potentialities exhibiting a TOF = 7596 h^–1^ similar to that observed in our standard conditions (8986 h^–1^; 7.5 μM) ([NAD^+^] = 2.0 mM, 313 K, phosphite buffer 0.4 M, pH 6.58). TON increases up to 1816, reaching the limit of the absorbance linearity region in UV–vis, with little sign of catalyst deactivation (Figure S28).

Albeit these kinetic results clearly indicate that H_2_PO_3_^–^ is a superior hydride donor, a more stringent comparison between the two H-donors was designed by performing catalytic experiments with **1** using H–COO^–^ and H–PO_3_H^–^ under exactly the same concentration (0.125 M), buffering the solution with phosphate (Figure 13). It was confirmed that phosphite is a better H-donor than formate; indeed, TOF was more than six times higher with the former (2521 versus 401 h^–1^) analogously to what was previously reported for [Cp*Ir­(6-NH_2_-pysa)­NO_3_], which exhibited a four times increase of TOF with phosphite.^18^

**Figure 13:**
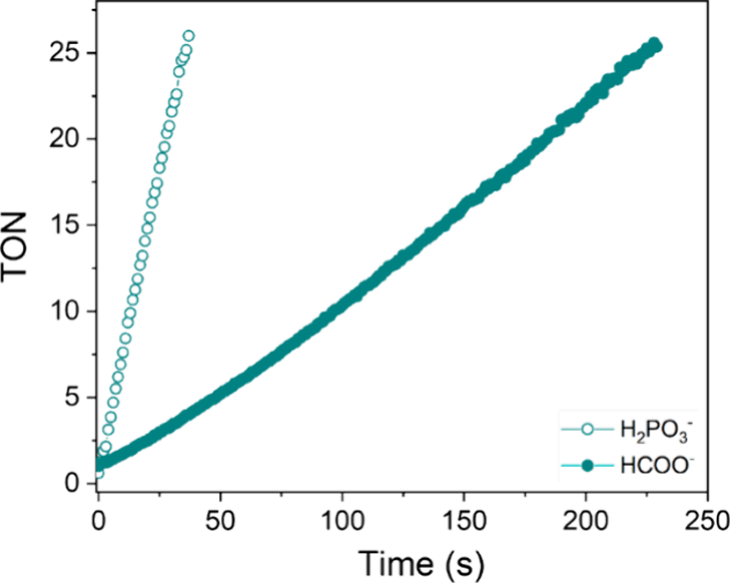
Time course of TON in the presence of phosphite or formate as hydride donors with **1**. ([cat] = 7.5 μM; [HCOO–/H_2_PO_3_−] = 0.125 M; phosphate buffer, pH 6.58 M, 313 K).

Kinetic data of catalyst **1** with H_2_PO_3_^–^ as a function of NAD^+^ concentration were nicely fitted using eq 2 (Figure S29) derived assuming the reaction mechanism shown in Figure 2:^18^

$$r_{\mathrm{NADH}}=\frac{k_{3}k_{2}[{DH}^{-}][\mathrm{Cat}][{\mathrm{NAD}}^{+}]}{k_{3}[{\mathrm{NAD}}^{+}]+k_{2}[{DH}^{-}]+K_{M}k_{3}[{\mathrm{NAD}}^{+}{]}^{2}}$$ 2

where *k*_2_ is the rate constant relative to the hydride formation step, *k*_3_ is the rate constant relative to the NAD^+^ hydrogenation step, and *K*_M_ is the binding constant for the formation of the inhibition adduct (**Ir_NAD** ^**+**^). Unfortunately, fittings of kinetic data of **2** and **3** were not accurate, likely because of the little accentuated maximum TOF, occurring at low [NAD^+^]. The kinetic and thermodynamic parameters found for **1** are compared to that of [Cp*Ir­(6-NH_2_-pysa)­NO_3_] in Table 4.

**Table 4:** Comparison of the Kinetic Constants Obtained by the Fitting of the Experimental Data of Catalyst **1** with Literature Values^18^ ^a^

	*k*_2_ (M^–1^ s^–1^)	*k*_3_ (M^–1^ s^–1^)	*K*_M_ (M^–1^)
**1**	23 ± 5	1889 ± 154	150 ± 62
[Cp*Ir(6-NH_2_-pysa)NO_3_]	6.40 ± 0.83	970 ± 40	191 ± 44

a Reaction conditions: [cat] = 7.5 μM; [H_2_PO_3_^–^/HPO_3_^2–^] = 0.4 M; pH = 6.58; *T* = 313 K for **1** and [cat] = 5.0 μM; [H_2_PO_3_^–^/HPO_3_^2–^] = 0.4 M; pH = 6.58; *T* = 313 K for [Cp*Ir­(6-NH_2_-pysa)­NO_3_].

Data shown in Table 4 clearly confirm that **1** is a better catalyst than [Cp*Ir­(6-NH_2_-pysa)­NO_3_] in all aspects. It forms **Ir_H** almost four times faster, indicating that the combined effect of less σ-donation and π-back-donation of pyrazine, with respect to pyridine, makes Ir even more acidic than when pysa is present. The tendency to donate the hydride is ca. 2-fold, whereas the tendency to form the off-cycle species **Ir_NAD** ^**+**^ is similar or somewhat smaller. This can be attributed to the inhibition preventive effect of the dangling NH_2_ in **1**, analogously to what was found for [Cp*Ir­(6-NH_2_-pysa)­NO_3_].^18,19^

#### NMR Experiments

The chemo- and regioselectivity of catalysts **1**–**3** toward the biologically active 1,4-NADH isomer was investigated by following the progress of the reaction by ^1^H NMR ([cat] = 100 μM, [NAD^+^] = 5 mM, phosphite buffer 0.4 M pH 6.58, 298 K) (Figure 14 and Figure S25).

**Figure 14:**
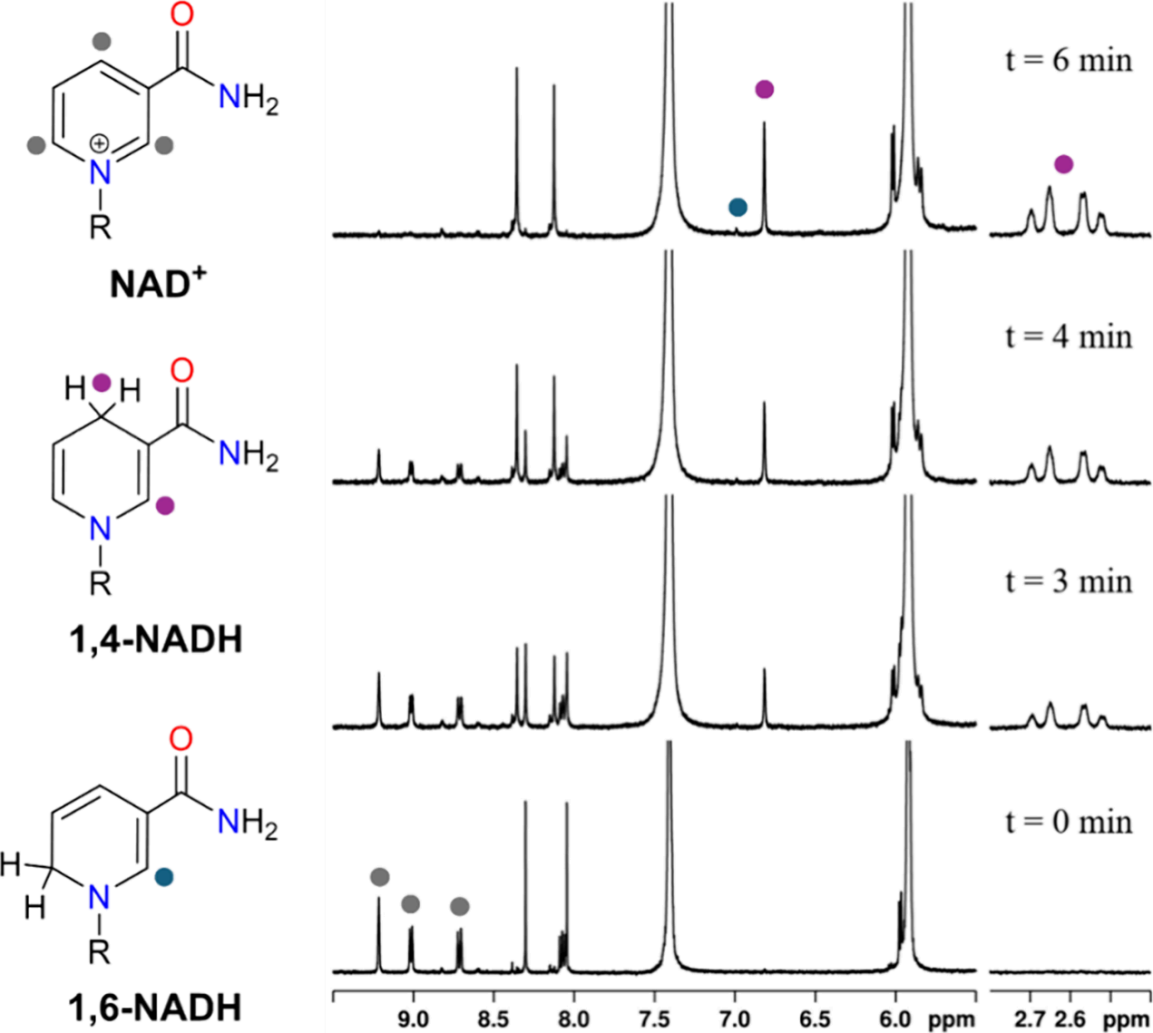
^1^H NMR spectra for the hydrogenation of NAD^+^ (gray) with phosphonic acid, catalyzed by **1**, showing the regioselective formation of 1,4-NADH (purple) and 1,6-NADH (blue) ([NAD^+^] = 5 mM, [cat] = 100 μM, buffer phosphite 0.4 M, pH = 6.58, *T* = 313 K).

NMR experiments evidenced the fast consumption of NAD^+^, whose resonances disappeared in 5, 20, and 60 min for **1**, **2**, and **3**, respectively, and main and clean formation of 1,4-NADH as indicated by the resonance at 2.6 ppm assigned to the diastereotopic CH_2_ protons in the 4-position of the pyridine ring (Figure 14). As previously reported by us, together with the typical signals of 1,4-NADH, a small percentage of 1,6-NADH (about 9% of total NADH) was observed owing to the ability of this class of catalysts to promote the reversible isomerization of 1,4 and 1,6-NADH.

It is important to outline that during all NMR experiments conducted in coordinating solvents as DMSO and CH_3_CN, in the presence of large excess of potentially coordinating anions (such as Cl^–^, HCOO^–^, H_2_PO_3_^–^), no sign of pyrazine detachment from the metal center. This is in sharp contrast with that found for [Cp*Ir­(6-NH_2_-pysa)­NO_3_] that, in order to reach the hydride intermediate, undergoes pyridine detachment from the metal center.^19^ For catalyst **1**, no evidence of the occurrence of a similar process was found, accordingly with the stronger electron-withdrawing properties of the pyrazine ring.

## 3 Discussion

The above results clearly demonstrated that the introduction of a less σ-donating and more π-accepting ligand, along with a basic and hindering functionality in proximity of the metal center, led to exceptional performances in NADH regeneration with catalyst **1**. These factors must be contemporarily present, as replacing the pyrazine ring with pyridine (**2**) or removing the −NH_2_ functionality (**3**) resulted in significantly lower catalytic activity. The main differences in the performance trends of these catalysts could be ascribed to a complex equilibrium between the rate of **Ir_H** formation (*k*_2_), which is likely the rate-determining step of the reaction, and the formation of the off-cycle adduct with NAD^+^. Catalyst **1** proved to have a remarkably enhanced metal acidity, which boosted the rate of the hydride formation step, at the same time limiting the tendency to form unproductive adducts with NAD^+^ (**Ir_NAD** ^**+**^). Catalyst **3**, on one side, is expected to have a similar metal acidity and a relatively similar tendency to form the hydride, but the absence of the hydrazide moiety lets the formation of off-cycle species have a prevailing effect on its catalytic properties. On the other side, the diminished metal acidity of the ligand limits the rate constant of the crucial step of the mechanism. The detrimental effect of the increased concentration of substrate on the activity of **2** and **3** can then be explained by their limited tendency to reach **Ir_H** and/or their pronounced tendency to form unproductive side species **Ir_NAD** ^**+**^. For **1**, with phosphite as the hydride donor, this trend was not observed since the binding of **Ir_H**_**2**_ **O** with NAD^+^ is expected to be significantly slower with respect to the P–H activation leading to **Ir_H** and NAD^+^ hydrogenation.

The balance of these two aspects is strictly dependent on the nature of the hydride donor employed for the reaction. We can reasonably assume that the tendency of **Ir_H**_**2**_ **O** to bind NAD^+^ does not depend on the nature of the hydride donor, so *K*_M_ should be the same when HCOO^–^ and H_2_PO_3_^–^ are employed. On the other hand, all catalysts displayed worst catalytic performances in the presence of formate. Notably, the maximum TOF achieved by catalyst **1** with formate was nearly 30 times lower than that in phosphite buffer. Intrinsically more difficult C–H versus P–H activation, higher coordination ability of HCOO^–^, and more marked tendency of H_2_PO_3_^–^ to act as proton shuttle^51^ are features that might explain the higher activity observed with phosphite. Additionally, the reduced catalytic activity exhibited with formate makes the formation of **Ir_NAD** ^**+**^ more significant and can explain the decreasing trend of the TOF as a function of the NAD^+^ concentration found for **1**.

Although it is always difficult to compare the activity of catalysts tested under different experimental conditions, the TOF attained by **1** (13,090 h^–1^) is really remarkable if compared with that of other organometallic compounds reported so far (Table 5).^52–54^ As shown, our catalysts clearly outperform the best Rh-based catalyst, [Cp*Rh­(phen)­Cl] (phen = phenantroline), for which a maximum TOF of 2000 h^–1^ has been obtained at 60 °C.^55^ **1** clearly exceeded the performances of all the iridium-based catalysts reported so far. Specifically, **1** was found to be almost four times more active than [Cp*Ir­(6-NH_2_pysa)­NO_3_], for which a TOF of 3731 h^–1^ was reported for UV–vis experiments with phosphite as the hydride donor. The activity of **1** is also higher than that of [Cp*Ir­(picaNphCOOH)­Cl], which was the fastest catalyst reported for this reaction so far.^16^ In addition, **1** demonstrated comparable or even superior activity to certain enzymes,^56,57^ for example, outperforming formate dehydrogenase in NADH regeneration under similar conditions (TOF = 9000 h^–1^).^57^ Furthermore, the catalyst we report here offers the significant benefits of homogeneous organometallic catalysts in terms of versatility and adaptability to various experimental conditions.

**Table 5:** TOF Values and Reaction Conditions of Some Catalysts for the Regeneration of NADH

catalyst	[cat] (μM)	[NAD^+^] (mM)	[DH^–^] (M)	pH	*T* (°C)	TOF (h^–1^)
[Cp*Rh(phen)Cl]^+^ ^52,53^	25	0.4	0.5^a^	7	38	81.5
[Cp*Rh(phen)Cl]^+^ ^54^	8	8	0.35^a^	7	60	2000
[Cp*Ir(5-NO_2_phen)Cl]^+^ ^54^	80	8	0.35^a^	7	38	58
[Cp*Ir(6-NH_2_pysa)NO_3_][Bibr ref18]	5	3	0.4^b^	6.58	40	3731
[Cp*Ir(pysaNphCOOH)Cl][Bibr ref58]	8	8	0.35^a^	6.5	37	1436
[Cp*Ir(pica)Cl][Bibr ref17]	7.5	0.77	0.125^a^	7	25	143
[Cp*Ir(picaNphCOOH)Cl][Bibr ref16]	0.8	8	0.35^a^	6.5	37	7825
**1**	7.5	6	0.4^b^	6.58	40	13090

a Formate (HCOO^–^) in phosphate buffer.

b Phosphite buffer (H_2_PO_3_^–^/HPO_3_^2–^).

## 4 Conclusions

A catalyst for the chemical regeneration of NADH, through the reaction of NAD^+^ with an inexpensive hydrogen donor such as phosphite, exhibiting performance superior to all other organometallic catalysts reported so far and comparable to that of enzymes, has been developed through a rational approach driven by a deep understanding of the reaction mechanism. The two elements of novelty, which determined the success of the design strategy, are the substitution of pyridine with pyrazine in the *N*,*N*-amidate bidentate supporting ligand and the presence of a carbohydrazide dangling groups. The pyrazine moiety has the double role of making the iridium center more acidic, being less σ-donating than pyridine, without weakening the Ir–N bond, being more π-accepting than pyridine. Those roles cause an increased tendency to activate the P–H bond of the donor and higher stability of the catalyst. The second element of novelty, i.e., the carbohydrazide dandling group in close proximity of the Ir center, likely facilitates the shuttling of the hydride from the donor to the metal, and from the metal to NAD^+^, and inhibits the formation of the detrimental, out-of-cycle **Ir_NAD** ^**+**^ adduct. Overall, the maximum TOF for **1** (13,090 h^–1^, [NAD^+^] = 6 mM, [cat] = 7.5 μM, pH 6.58 by phosphite buffer 0.4 M, 313 K) is remarkable and bodes well that this catalyst can actually be used in NADH regeneration processes of practical interest.

## 5 Experimental Section

### Materials and Methods

All reagents and organic solvents were purchased from Sigma-Aldrich and used as received. Water was purified by using a Milli-Q Ultrapure water purification system. [Cp*IrCl_2_]_2_ was prepared according to the literature.^59^ NMR spectra were recorded on a Bruker Avance III 400 spectrometer equipped with a SmartProbe (400 MHz for 1H) and a *z* gradient coil. Residual solvent resonances were used for referencing; reported chemical shifts are relative to those of external TMS. X-ray diffraction patterns of the single crystal of complexes **1**_**D**_, **2**, and **2**_**D**_ were recorded at 298 K using a Bruker D8 Venture diffractometer equipped with an Incoatec ImuS3.0 microfocus sealed-tube MoKα (λ = 0.71073 Å) source and a CCD Photon II detector. The data, collected through generic φ and ω scans, were integrated and reduced using the Bruker AXS V8 Saint Software. The structure was solved and anisotropically refined using the SHELXT and SHELXL packages of the Bruker APEX3 software.5 HR-MS experiments were performed on an Agilent 6540 time-of-flight (Q-TOF) system, operated in MS mode, with a Dual AJS ESI source that operated positive with N_2_ as desolvation gas.

The diffusion coefficient of the iridium complexes (*D*_cat_) was measured by ^1^H PGSE (pulsed-field gradient spin echo) NMR experiments at 298K using the double-simulated echo pulse sequence. A series of ^1^H NMR spectra were recorded by varying the strength of the pulsed-field gradient (*G*) along the *z* axis. The diffusion time (Δ) was set to 90 ms, and the length of the gradient pulse (δ) was constant and equal to 5 ms. Signal intensities, *I*, were measured by integration. The plot of ln­(*I*/*I*_0_), where *I*_0_ represents the intensity of resonance in the absence of the pulsed-field-gradient, against *G* ^2^ gives a linear correlation. The slope of the linear regression (*m*) is proportional to the diffusion coefficient *D* according to eq 3:^60^

$$\ln\;\frac{I}{I_{0}}=mG^{2}\;\mathrm{in}\;\mathrm{which}\;m={\left(\delta \gamma \right)}^{2}D\left(\Delta -\frac{\delta }{3}\right)$$ 3

where γ is the gyromagnetic ratio of ^1^H.

Magnetization transfer rate constants were measured by two-dimensional ^1^H EXSY NMR experiments acquired with a matrix of 1024 × 512 data points and a mixing time of 800 ms. The raw data were processed using zero-filling to obtain 2048 data points in both spectral dimensions. Magnetization transfer rate constant values were determined as described in the literature^61^ using the EXSY CALC software.^62^

### General Procedure for the Regeneration of NAD^+^

The reaction was monitored by UV–vis spectroscopy, following the formation of the absorption band of NADH at 340 nm (ε = 6220 M^–1^ cm^–1^), using a diode array spectrometer (HP 8453). In a typical experiment, a solution of NAD^+^ (3 mL) in phosphite buffer (pH = 6.58, 0.4 M) or phosphate buffer (pH = 7, 0.1 M, containing 0.125 M HCOOK) was transferred to a cuvette. The system was allowed to equilibrate under stirring for 10 min at 313 K; after the background correction, 15–30 μL of a stock solution of the catalyst in acetonitrile (2–3 mM) was injected and acquisition started. Taking into account the above-reported ε, the formation of NADH could be quantitatively evaluated only up to ca. 0.16 mM (linear region of the Lambert–Beer equation).

#### Synthesis of Pyza-NH_2_

One equivalent of 2-pyrazinamide (2.5 g, 20.1 mmol) and 3 equiv of hydrazine (1.95 mL, 60.9 mmol) were dissolved in 25 mL of ethanol under stirring. The solution was refluxed for 48 h, during which the reaction product precipitated as a white solid. It was subsequently recovered by filtration and washed three times with cold ethanol. Yield = 59%. ^1^H NMR (400 MHz, (CD_3_)_2_SO, 298 K, δ in ppm): δ = 10.13 (s,H4), 9.12 (d, ^4^ *J*_HH_ = 1.47, H3), 8.83 (d, ^3^ *J*_HH_ = 2.49, H1), 8.69 (dd, ^3^ *J*_HH_ = 2.49, ^4^ *J*_HH_ = 1.47, H2), 4.64 (s, H5).

#### Synthesis of Ethyl Pyridine-2-carboxylate

One equivalent of 2-picolinic acid (10 g, 81 mmol) and 0.35 equiv of sulfuric acid (2 mL, 28 mmol) were dissolved in 120 mL of ethanol under stirring. The solution was refluxed for 18 h, and the solvent was removed under vacuum. The ethyl pyridine-2-carboxylate so obtained was dissolved in 30 mL of methylene chloride and washed two times with a 5% wt. aqueous solution of NaHCO_3_, two times with Milli-Q and anhydrified with MgSO_4_. The solvent was then removed under vacuum, and the product was recovered as a dense white liquid. Yield = 50%. ^1^H NMR (400 MHz, (CD_3_)_2_SO, 298 K, δ in ppm): δ = 8.74 (m, H4), δ = 8.05 (m, H3, H2), δ = 7.68 (m, H1), δ = 4.36 (q, ^3^ *J*_HH_ = 7.13, H5), δ = 1.34 (t, ^3^ *J*_HH_ = 7.13, H6).

#### Synthesis of Pica-NH_2_

One equivalent of ethyl pyridine-2-carboxylate (6.1 g, 40 mmol) and 3 equiv of hydrazine monohydrate (120 mmol) were dissolved in 20 mL of ethanol and refluxed for 24 h. The solvent was removed under vacuum, and the product was recovered as a white powder and washed with cold ethanol. Yield = 61%. ^1^H NMR (400 MHz, (CD_3_)_2_SO, 298 K, δ in ppm): δ = 9.86 (b, H5), 8.61­(dt, ^3^ *J*_HH_ = 4.75, ^4^ *J*_HH_ = 1.33, H4), 7.98 (m, H1, H3), 7.57 (m, H2), 4.56 (d, ^3^ *J*_HH_ = 4.03, H6).

#### Synthesis of **1**

One equivalent of [Cp*IrCl_2_]_2_ (0.1 g, 0.125 mmol) was suspended in 5 mL of methanol. 2.1 equivalents of pyza-NH_2_ (36 mg, 0.26 mmol) were dissolved in 5 mL of methanol and added to the starting solution, which turns red. This solution was reacted with 10 mL of methanol containing 2.1 equiv of KOH (9.25 mg, 0.26 mmol) and stirred for 6 h. After the solvent was removed under low pressure, the product was dissolved in CH_2_Cl_2_, filtered to remove residual KCl, and dried under vacuum. Exp: C 36.08% (36.03% th) H 4.15% (4.03% th) N 11.19% (11.21% th). Yield = 65%. ^1^H NMR (600 MHz, (CD_3_)_2_SO, 298 K, δ in ppm): δ = 8.89 (d, ^4^ *J*_HH_ = 1.19, H5), 8.75 (dd, ^3^ *J*_HH_ = 3.12, ^4^ *J*_HH_ = 1.19, H3), 8.71 (d, ^3^ *J*_HH_ = 3.12, H4), 5.17 (b, H8), 1.71 (s, H1). ^13^C {^1^H} NMR (150 MHz, (CD_3_)_2_SO, 298 K, δ in ppm): δ = 162.4 (C7), δ = 147.6 (C4), δ = 147.5­(C6), δ = 145.6 (C5), δ = 144.8 (C3), δ = 88.2 (C2), δ = 8.9 (C1).

#### Synthesis of **1_D_**

87.61 mg of **1** (0.165 mmol) was dissolved in 5 mL of MeOH and reacted with about 20 equivalents of NH_4_PF_6_ solid under stirring. The instantaneous precipitation of **1**_**D**_ as a yellow solid was observed. The solid was recovered by filtration and washed with MeOH (2 × 1 mL) and diethyl ether (3 × 5 mL). Exp: C 29.12% (29.56% th) H 3.63% (3.31% th) N 11.19% (11.07% th). Yield = 42%. ^1^H NMR (400 MHz, (CD_3_)_2_SO, 298 K, δ in ppm): δ = 9.09 (d, ^4^ *J*_HH_ = 1.17, H5), 8.89 (d, ^3^ *J*_HH_ = 3.14, H4), 8.80 (dd, ^3^ *J*_HH_ = 3.14, ^4^ *J*_HH_ = 1.17, H3), 5.38 (s, H8), 1.76 (s, H1). ^13^C {^1^H} NMR (100 MHz, (CD_3_)_2_SO, 298 K, δ in ppm): δ = 164.4 (C7), δ = 149.8 (C5), δ = 147.5 (C6), δ = 146.4 (C4), δ = 146.1 (C3), δ = 96.6 (C2), δ = 8.5 (C1).

#### Synthesis of **2**

One equivalent of [Cp*IrCl_2_]_2_ (80 mg, 0.1 mmol) was suspended in 5 mL of methanol. 2.1 equivalents of pica-NH_2_ (29 mg, 0.21 mmol) were dissolved in 5 mL of methanol and added to the starting solution, which turns red. This solution was reacted with 10 mL of methanol containing 2.1 equiv of NaOH (8.4 mg, 0.21 mmol) and stirred for 6 h. After the solvent was removed under low pressure, the product was dissolved in CH_2_Cl_2_, filtered to remove residual KCl, and dried under vacuum. Yield = 65%. Exp: C 38.87% (38.51% th) H 4.39% (4.24% th) N 8.36% (8.42% th). ^1^H NMR (600 MHz, (CD_3_)_2_SO, 298 K, δ in ppm): δ = 8.65 (d, ^3^ *J*_HH_ = 5.34, H6), 8.03 (m, H4), 7.74 (d, ^3^ *J*_HH_ = 7.73, H3), 7.55 (m, H4), 5.14 (b, H8), 1.69 (s, H10). ^13^C {^1^H} NMR (150 MHz, (CD_3_)_2_SO, 298 K, δ in ppm): δ = 151.1 (C6), 139.5 (C4), 126.8 (C5), 123.8 (C3), 87.1 (C9), 9.0 (C10).

#### Synthesis of **2_D_**

82 mg of **2** (0.159 mmol) was dissolved in 5 mL of MeOH and reacted with about 20 equivalents of NH_4_PF_6_ solid under stirring. The instantaneous precipitation of **2**_**D**_ as a yellow solid was observed. The solid was recovered by filtration and washed 2 × 1 mL MeOH and 3 × 5 mL diethyl ether. Yield = 40%. Exp: C 31.09% (31.58% th) H 3.55% (3.48% th) N 6.76% (6.91% th). ^1^H NMR (600 MHz, (CD_3_)_2_SO, 298 K, δ in ppm): δ = 8.72 (d, ^3^ *J*_HH_ = 5.32, H6), 8.29 (m, H4), 7.97 (d, ^3^ *J*_HH_ = 7.66, H3), 7.76 (m, H5), 5.28 (b, H8), 1.76 (s, H10). ^13^C {^1^H} NMR (150 MHz, (CD_3_)_2_SO, 298 K, δ in ppm): δ = 165.8 (C7), δ = 152.9 (C2), δ = 152.8 (C6), δ = 142.0 (C4), δ = 129.2 (C5), δ = 126.0 (C3), δ = 95.9 (C9), δ = 8.5 (C10).

## Supplementary Material



## Notes

Additional NMR spectra, semilogarithmic diffusion plots, and supplementary crystallographic data for **1**, **2**, **1**_D_, and **2**_**D**_ complexes (PDF)
