# Supplementary material for: Organometallic Catalysis Catches up with Enzymatic in the Regeneration of NADH
Source: ACS Catal. 2025 May 19;15(11):9417–29. doi: 10.1021/acscatal.5c02162 (PMC12150521; doi:10.1021/acscatal.5c02162)
Supplement: Supplementary file 1 [file cs5c02162_si_001.pdf]

Supporting Information.

Organometallic Catalysis Catches up with Enzymatic in the Regeneration of NADH

Caterina Trotta,[a] Giuseppe Fraschini,[a] Elena Tacchi,[b] Leonardo Tensi,[c] Cristiano Zuccaccia,[a] Gabriel Menendez Rodriguez,[a] and Alceo Macchioni[a]**

[a] Department of Chemistry, Biology and Biotechnology and CIRCC, University of Perugia, Via Elce di Sotto, 8-06123 Perugia, Italy;

[b] Department of Chemical Sciences, University of Padova, via Marzolo 1-35131 Padova, Italy

[c] Department of Pharmaceutical Science, University of Perugia, Via del Liceo 1- 06123 Perugia, Italy;

Email*: gabriel.menendezrodriguez@unipg.it alceo.macchioni@unipg.it

Table of Contents

NMR characterization of complexes 2

Cristallographic Characterization of complexes 11

Catalytic and Kinetic measurements 13

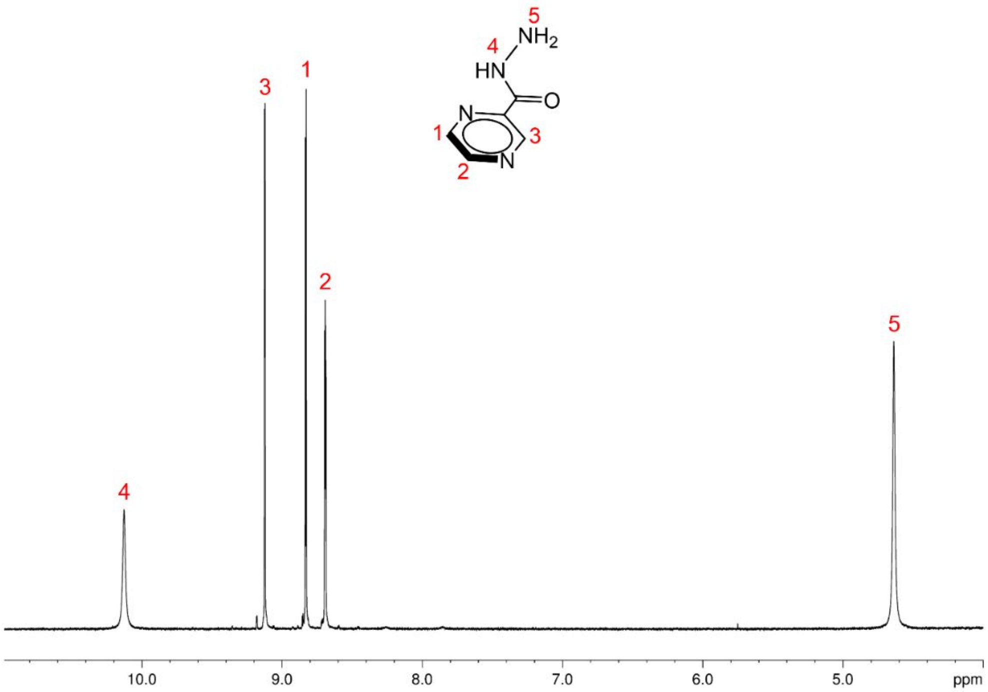

Figure S1. ^{1}H NMR spectrum of pyza–NH_2 $(CD_3)_2SO$, 298 K.

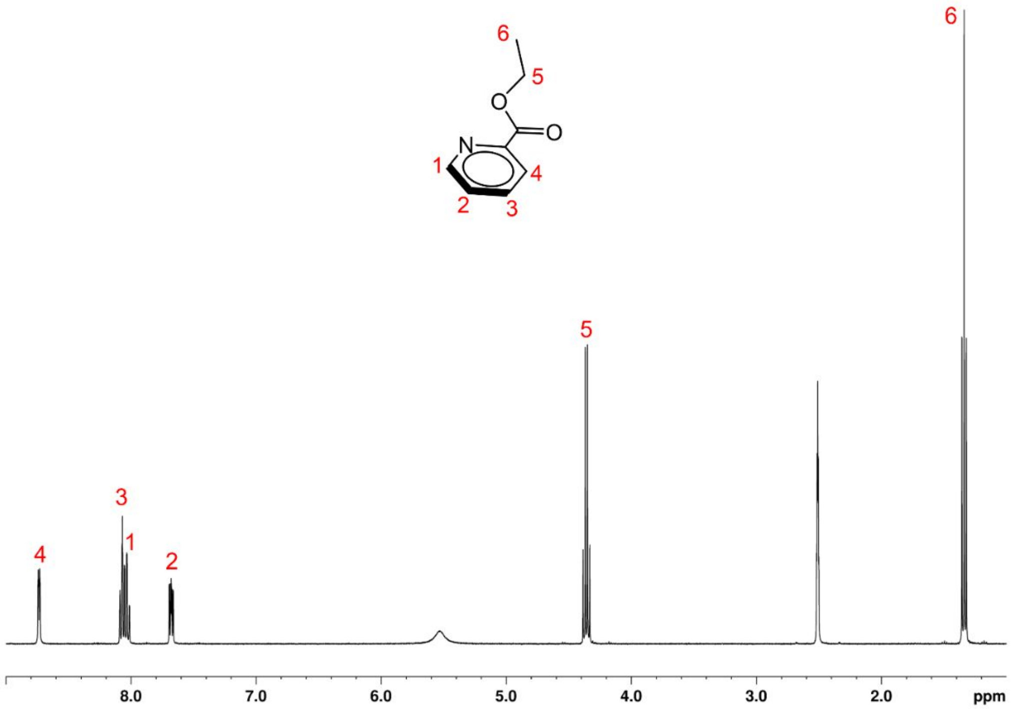

Figure S2. ^{1}H NMR spectrum of ethyl pyridine-2-carboxilate $(CD_3)_2SO$, 298 K.

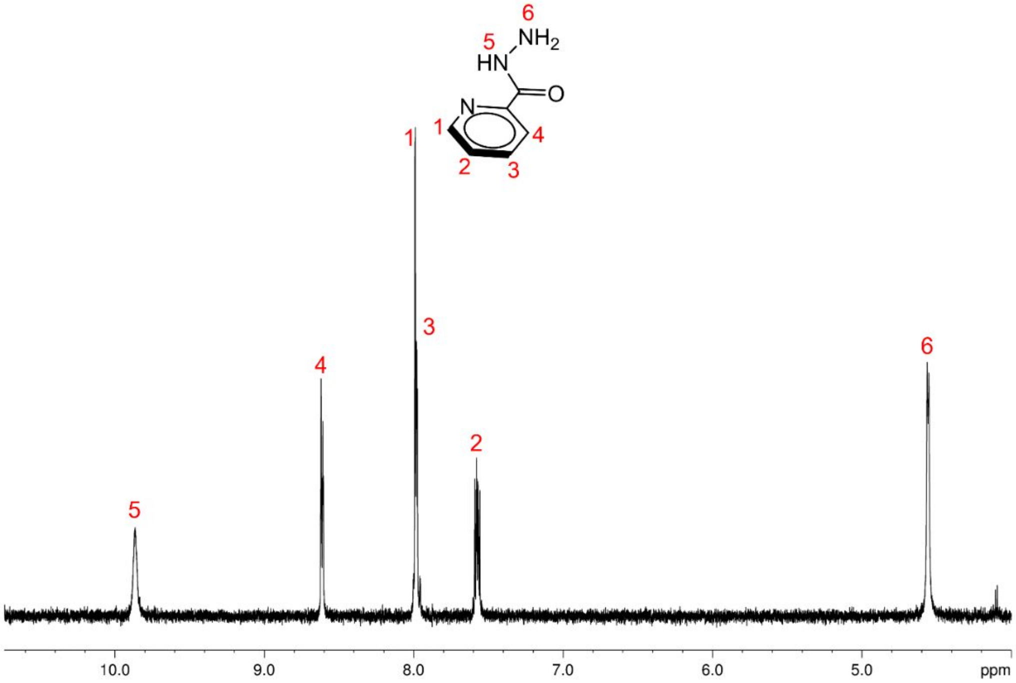

Figure S3. ^{1}H NMR spectrum of pica–NH_2 ($(CD_3)_2SO$, 298 K).

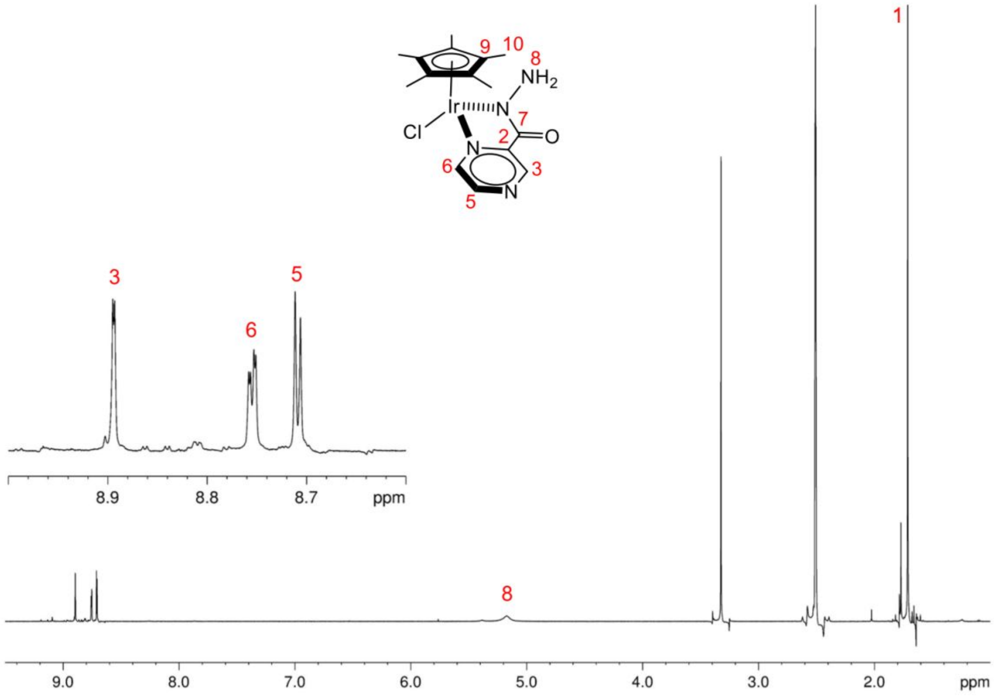

Figure S4. ^{1}H NMR spectrum of **1** $(CD_3)_2SO$, 298 K

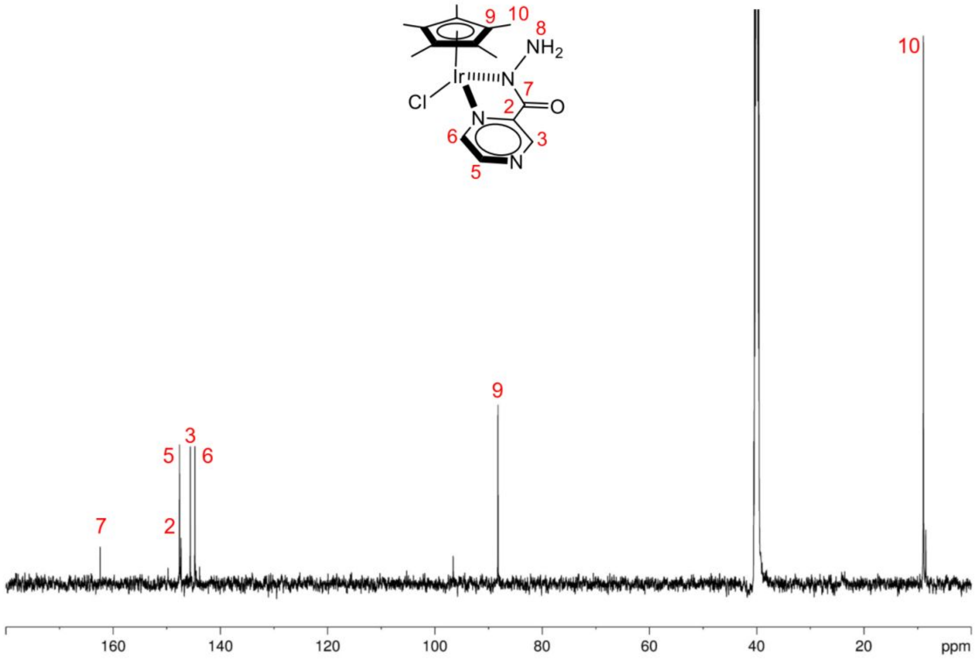

Figure S5. ^{13}C NMR spectrum of **1** $(CD_3)_2SO$, 298 K.

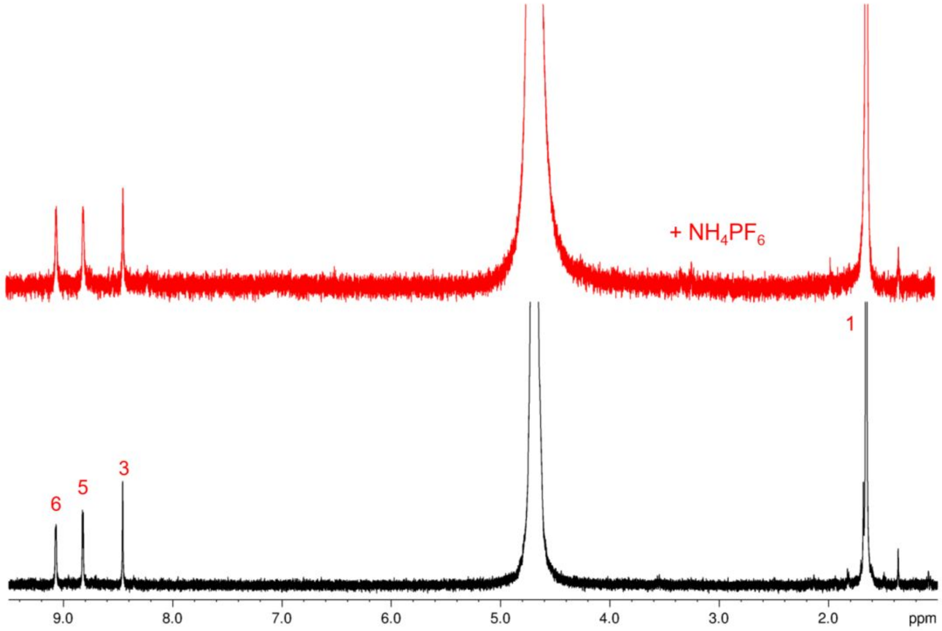

Figure S6. Stacked ^{1}H NMR spectra of **1** in D_2O (black) and after the addition of an excess of NH_4PF_6 (red).

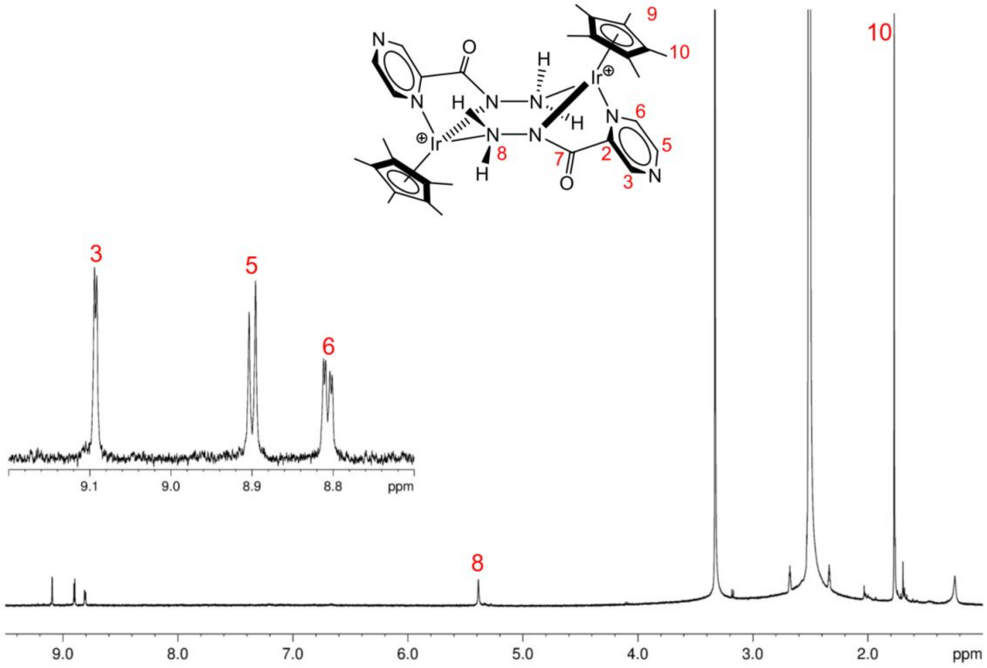

Figure S7. ^{1}H NMR spectrum of $\mathbf{1_D}$ $(CD_3)_2SO$, 298 K

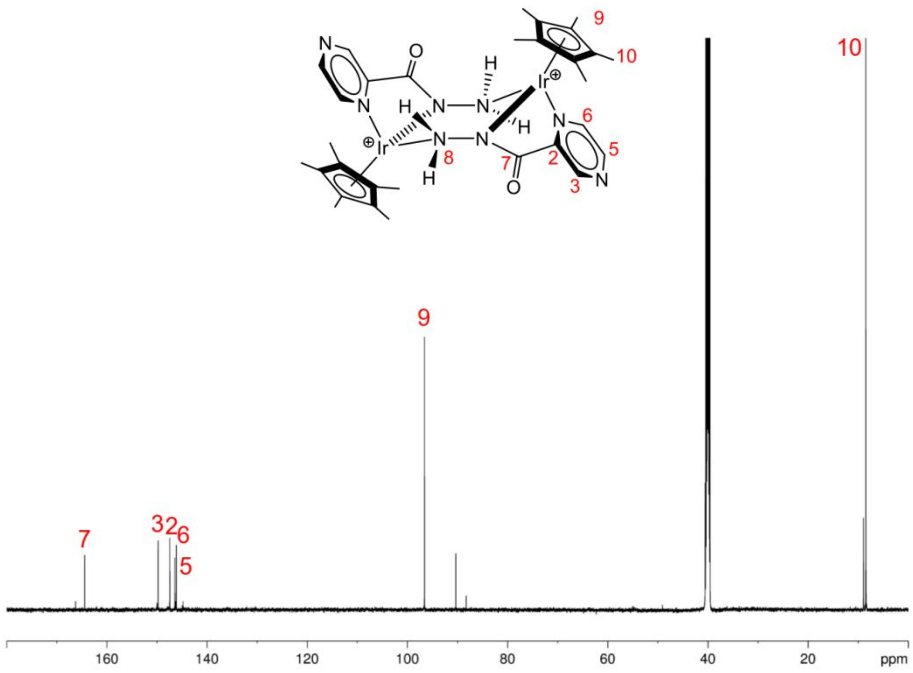

Figure S8. ^{13}C NMR spectrum of $\mathbf{1_D}$ $(CD_3)_2SO$, 298 K

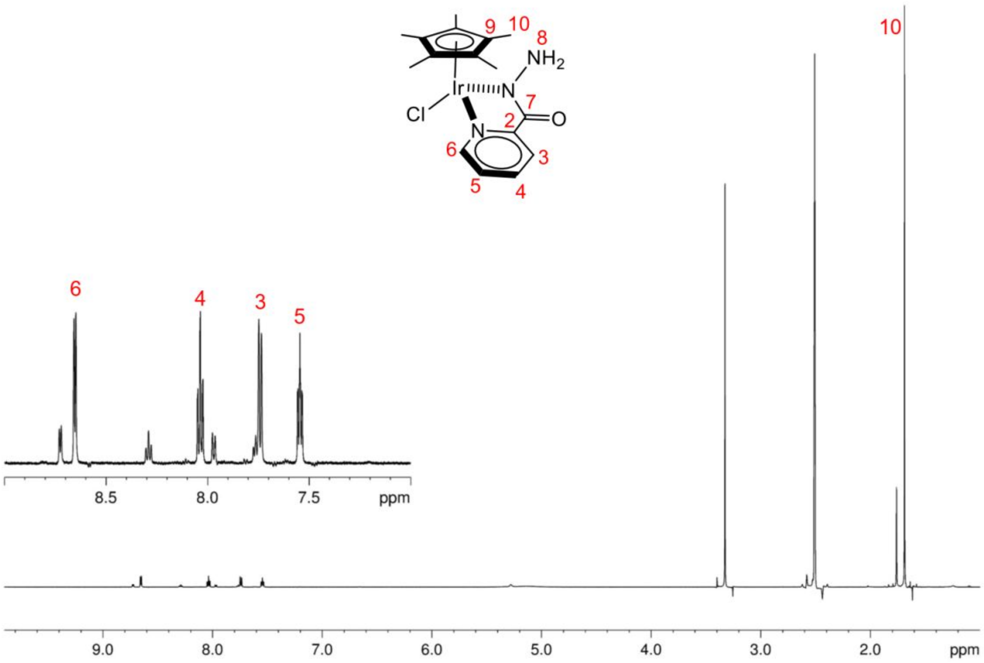

Figure S9. ^{1}H NMR spectrum of **2** $(CD_3)_2SO$, 298 K.

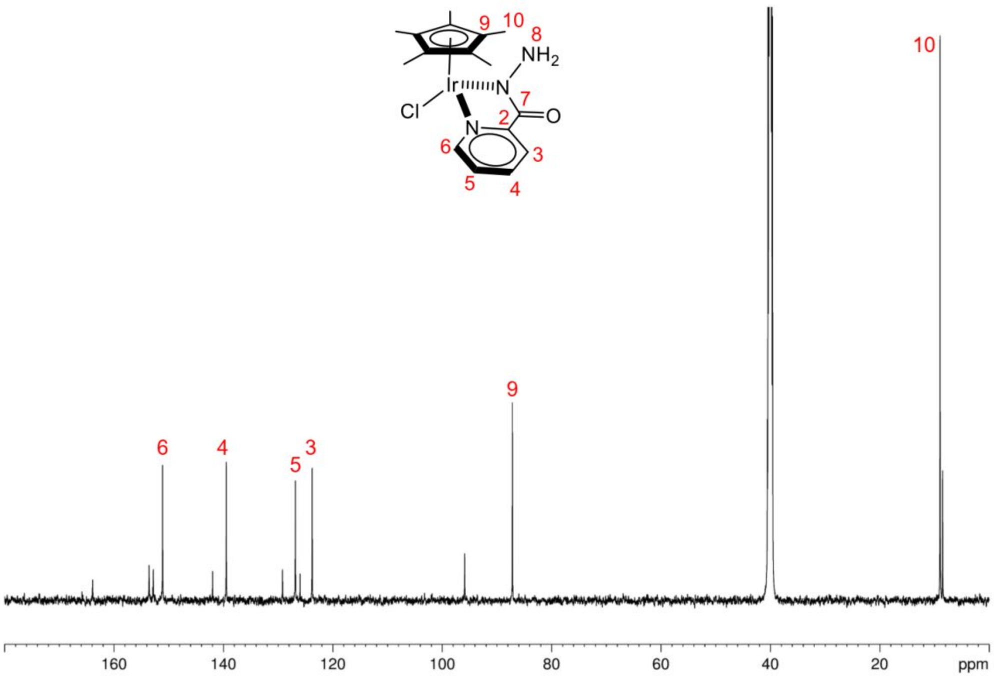

Figure S4.10. ^{13}C NMR spectrum of **2** $(CD_3)_2SO$, 298 K.

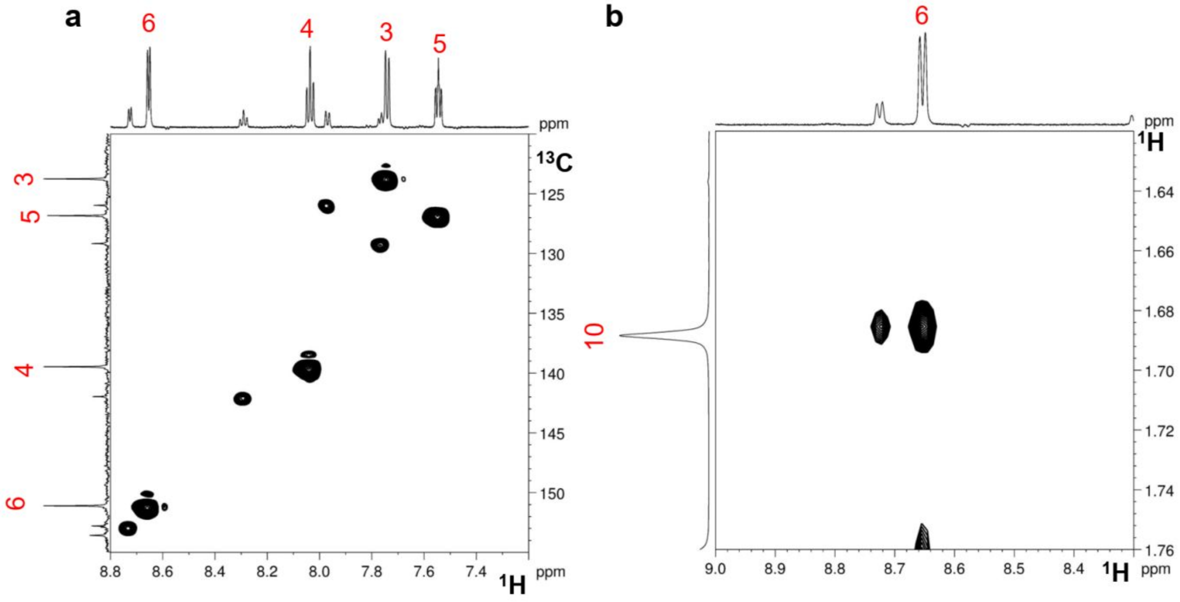

Figure S11. a) Section of the $^{13}C,^{1}H$ HSQC spectrum of **2** showing the scalar correlation of the aromatic protons b) section of the ^{1}H NOESY spectrum of **2** showing the dipolar correlation of H6 with H10 in $(CD_3)_2SO$, 298 K. Minor resonances are attributed to the formation of $\mathbf{2}_{D.}$

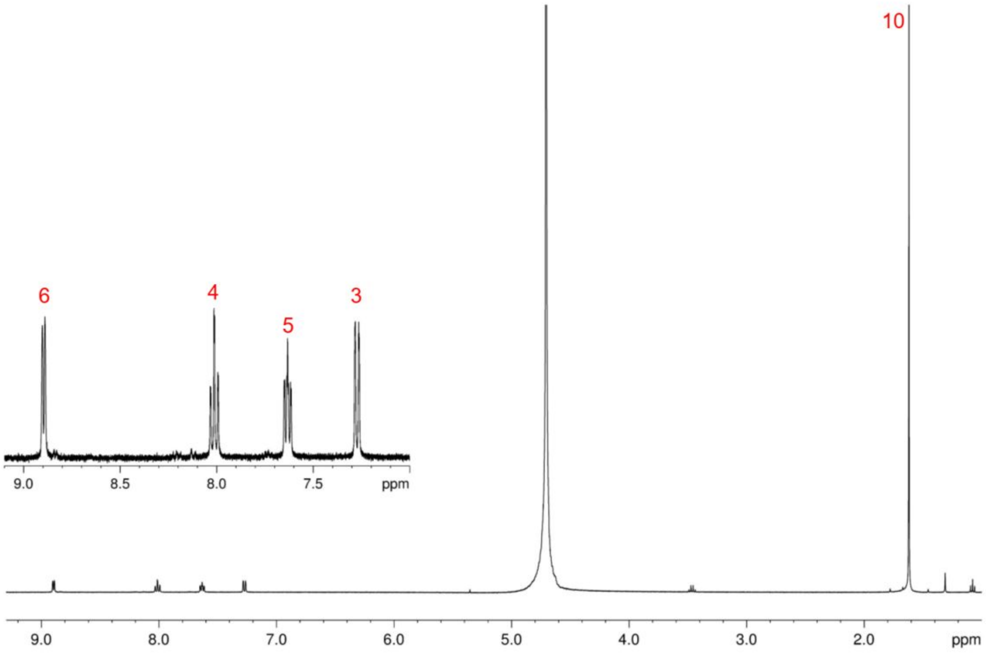

Figure S12. ^{1}H NMR spectrum of **2** D_2O, 298 K.

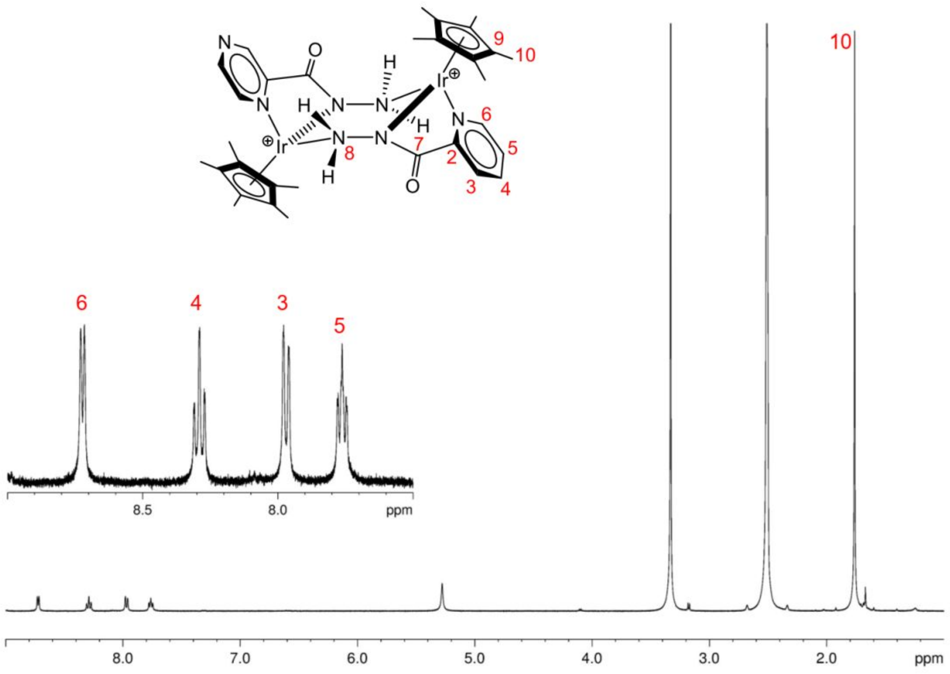

Figure S13. ^{1}H NMR spectrum of $\mathbf{2_D}$ $(CD_3)_2SO$, 298 K.

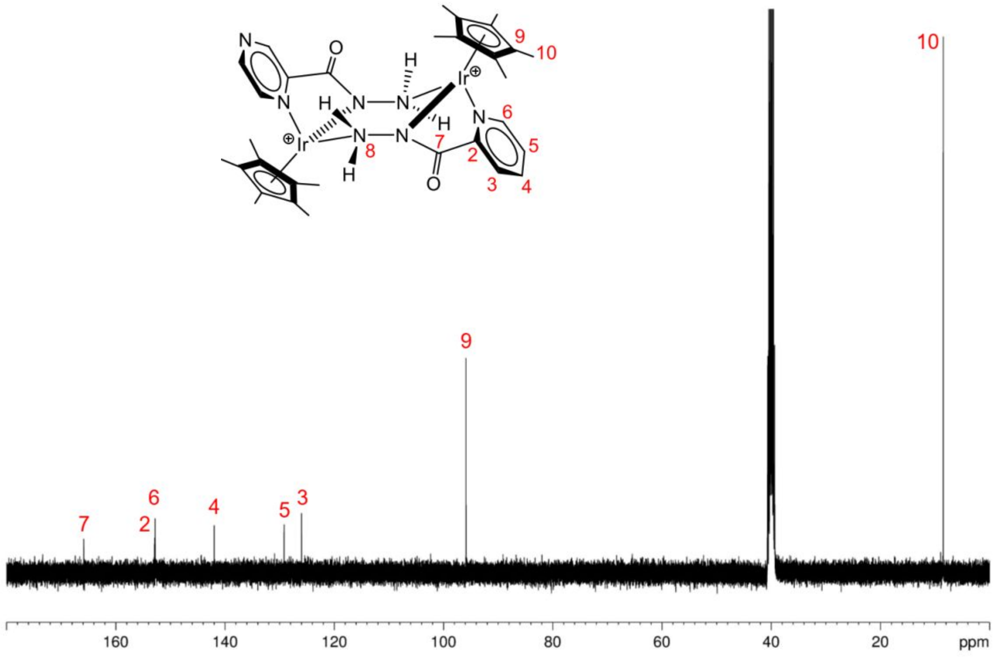

Figure S14. ^{13}C NMR spectrum of $\mathbf{2_D}$ $(CD_3)_2SO$, 298 K.

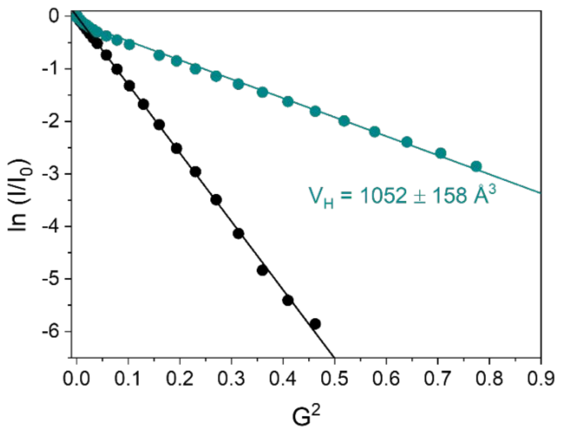

Figure S15. Semilogarithmic plots of $\ln(I/I_0)$ versus G^2 for $\mathbf{1_D}$ (blue) in DMSO-d_6 (black)

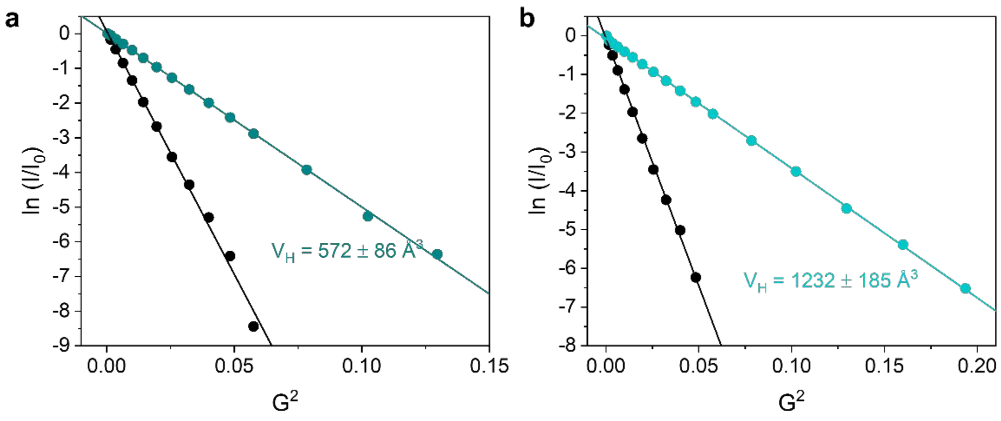

Figure S16. a) Semilogarithmic plots of $\ln(I/I_0)$ versus G^2 for **1** (green) in CD_3CN (black) b) Semilogarithmic plots of $\ln(I/I_0)$ versus G^2 for $\mathbf{1_D}$ (blue) in CD_3CN (black).

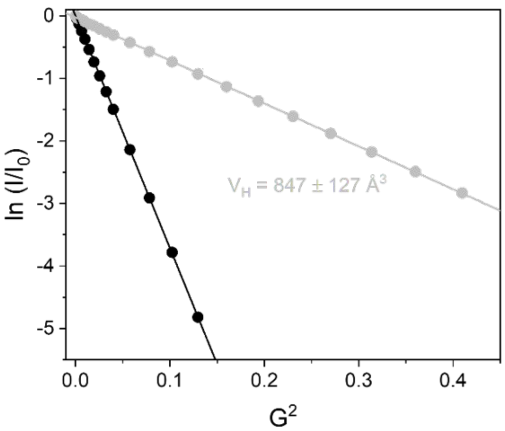

Figure S17. Semilogarithmic plots of ln(I/I_0) versus G^2 for $\mathbf{2_D}$ (grey) in D_2O (black).

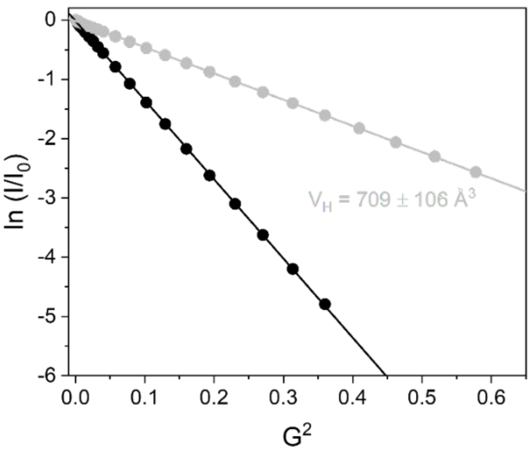

Figure S18. Semilogarithmic plots of ln(I/$_{I0}$) versus G^2 for $\mathbf{2_D}$ (grey) in DMSO-d_6 (black).

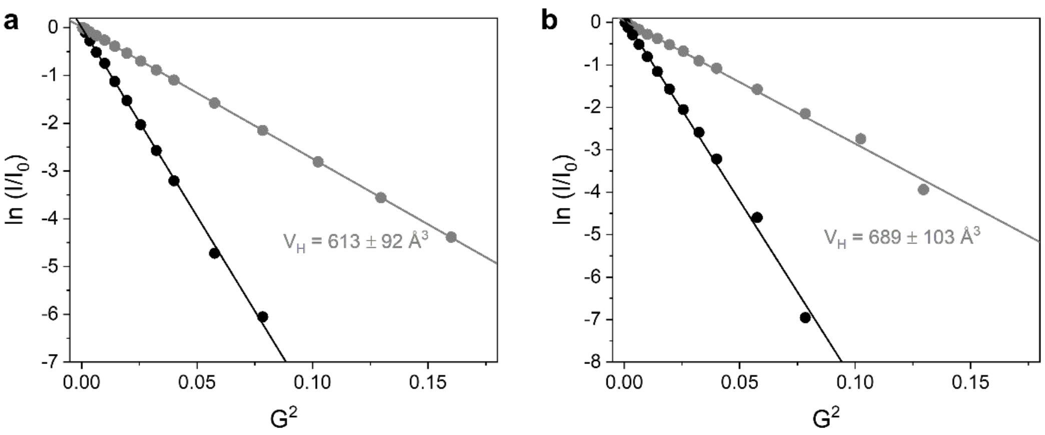

Figure S19. a) Semilogarithmic plot of $\ln(I/I_0)$ versus G^2 for **2** (grey) in CD_3CN (black). b) Semilogarithmic plot of $\ln(I/I_0)$ versus G^2 for **2** (grey), obtained from the dissolution of $\mathbf{2_D}$ in CD_3CN (black).

Table ES 1. Crystallographic data for **2**, **2_D** and **1_D**

	2	2_D	1_D
Empirical Formula	$C_{17}H_{25}IrN_3Cl$	$C_{16}H_{21}F_6IrN_3OP$	$C_{15}H_{20}F_6IrN_4OP$
Formula Weight	499.01	608.53	609.53
Crystal System	monoclinic	monoclinic	monoclinic
Space Group	P 2(1)/n	C 1 (2)/c1	C 1 (2)/c1
Unit Cell Dimensions	a = 10.138(2) Å<br>b = 15.477 (4) Å<br>c = 11.269 (2) Å<br>β = 107.619 (1)°	a = 22.409(15) Å<br>b = 9.338(6) Å<br>c = 19.506 (11) Å<br>β = 108.494(2)°	a = 22.710(5) Å<br>b = 9.051(2) Å<br>c = 19.765(4) Å<br>β = 111.119(8)
Volume	1685.26(6) Å^3	3870.8(4) Å^3	3789.8(15) Å^3
Z	4	8	8
Density (calculated)	1.967 g cm^{-3}	2.088 g cm^{-3}	2137 g cm^{-3}
Absorption coefficient	8.086 mm^{-1}	7.049 mm^{-1}	7.202 mm^{-1}
F(000)	960	2336	2336
Crystal size	0.080 x 0.060 x 0.020 mm^3	0.160 x 0.160 x 0.080 mm^3	0.080 x 0.080 x 0.040 mm^3
Theta range for data collection	2.31 to 28.33°	2.49 to 28.29°	2.54 to 28.29°
Index ranges	-13 ≤ h ≤ 12	-29 ≤ h ≤ 29	-30 ≤ h ≤ 29

	-20 ≤ k ≤ 20	-10 ≤ k ≤ 12	-12 ≤ k ≤ 12
	-14 ≤ l ≤ 15	-21 ≤ l ≤ 26	-26 ≤ l ≤ 25
Reflections collected	14803	71604	32529
Independent reflections	4188 [R(int) = 0.0409]	4775 [R(int) = 0.0288]	4606 [R(int) = 0.0315]
Completeness	to theta 28.28°,	to theta 28.29°,	to theta 28.29°,
	99.6%	99.3%	97.5%
Absorption correction	Multi-Scan	Multi-Scan	Multi-Scan
Max. and min. transmission	0.5849 and 0.7457	0.5409 and 0.7457	0.6243 and 0.7457
Data / restraints / parameters	4188/669/306	4775/0/258	4606/0/258
Goodness-of-fit on F2	1.030	1.104	1.048
Final R indices [I>2sigma(I)]	R1 = 0.0278	R1 = 0.0155	R1 = 0.0164
	wR2 = 0.0509	wR2 = 0.0346	wR2 = 0.0361
R indices (all data)	R1 = 0.0411	R1 = 0.0176	R1 = 0.0199
	wR2 = 0.0544	wR2 = 0.0356	wR2 = 0.0388
Largest diffraction peak and hole	0.940 and 1.117 Å	0.488 and 0.875 Å	0.699 and 0.454 Å

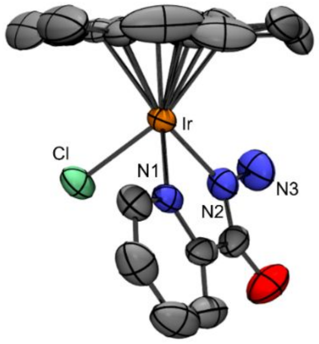

Figure S20. Ortep drawing of complex 2. Ellipsoid at 50% of probability, hydrogen atoms are omitted for clarity. Color code: Ir = orange, N = blue, O = red, C = grey and Cl = green. Relevant distances (Å) and angles (deg): Ir−Cp* = 1.770, Ir−Cl = 2.402, Ir−N1 = 2.110, Ir−N2 = 2.057, N2-N3 = 1.427, N1−Ir−N2 = 75.75, Ir-N2-N3 = 123.68, Cp*−Ir−N2 = 130.13, and Cp*−Ir−Cl = 129.02.

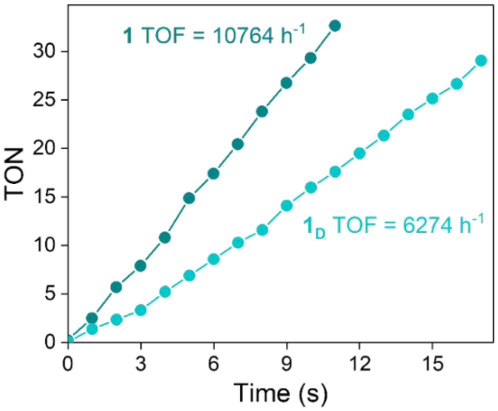

Figure S21. TON *vs.* time for two separate kinetic experiments made with **1** and $\mathbf{1_D}$ as starting complex. ([cat] = 7.5 mM; [NAD^+] = 5.0 mM; [$H_2PO_3^-/HPO_3^{2-}$] = 0.4 M; pH = 6.58; T = 313 K)

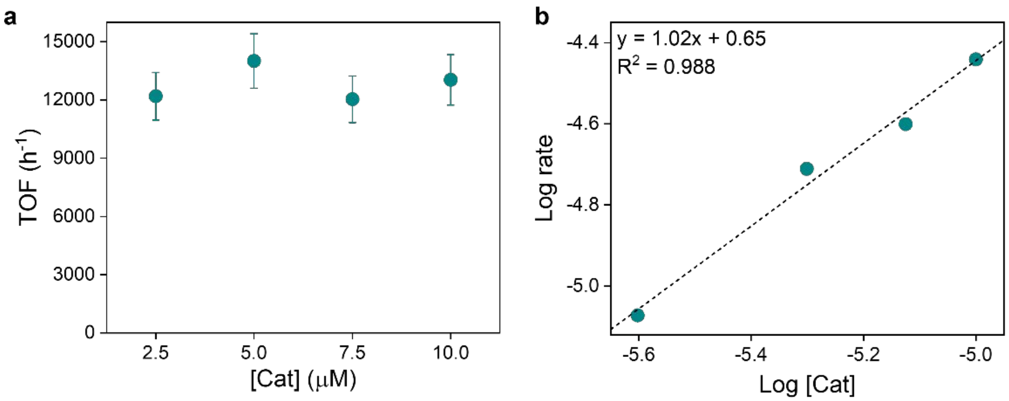

Figure S22. TOF *vs.* [cat] for **1** ([NAD+] = 5 mM, phosphite buffer 0.4 M pH 6.58, 313 K) (a). Log rate *vs.* Log [cat] plot.

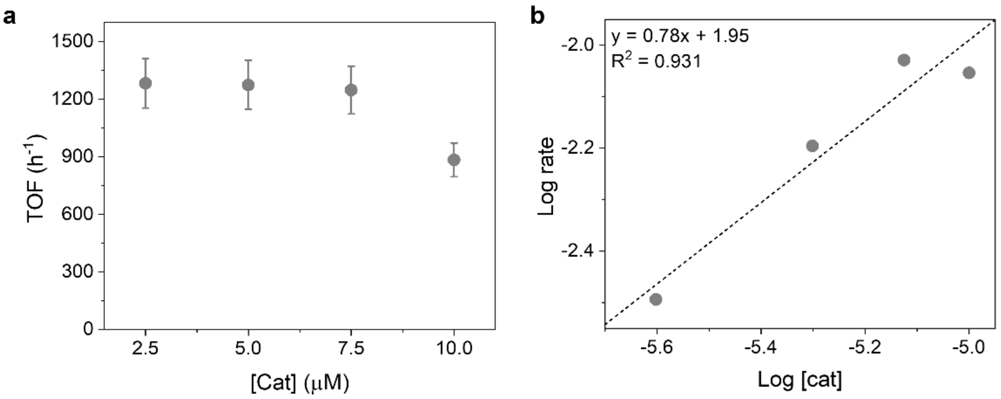

Figure S23. TOF *vs.* [cat] for **2** ([NAD+] = 0.5 mM, phosphite buffer 0.4 M pH 6.58, 313 K) (a). Log rate *vs.* Log [cat] plot.

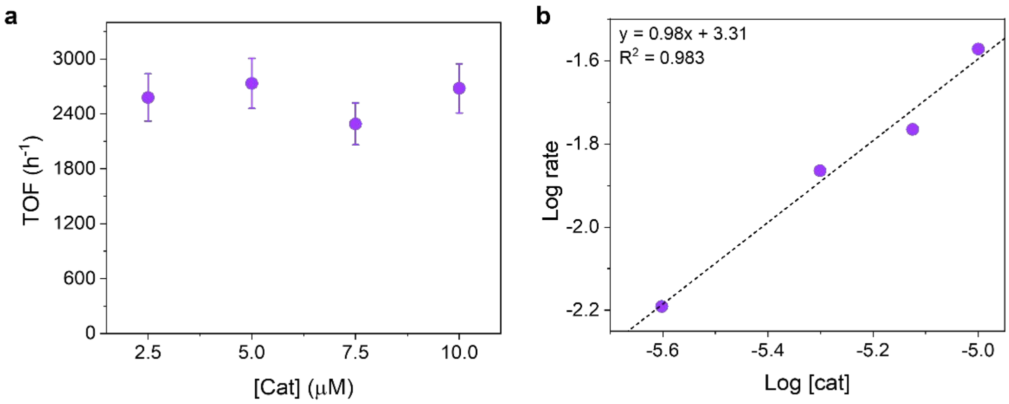

Figure S24. TOF *vs.* [cat] for **3** ([NAD+] = 1 mM, phosphite buffer 0.4 M pH 6.58, 313 K) (a). Log rate *vs.* Log [cat] plot.

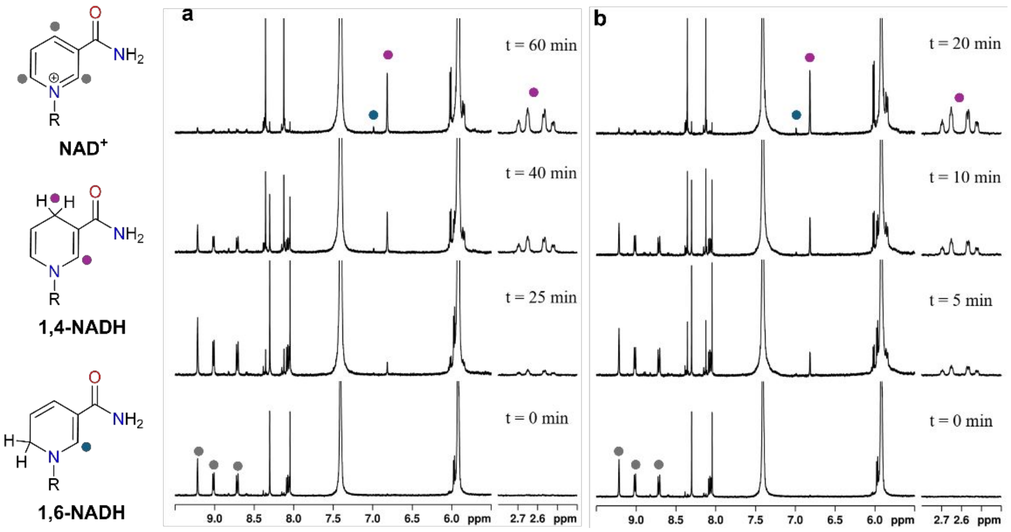

Figure S25. ^1H NMR spectra for the hydrogenation of NAD^+ (grey) with phosphonic acid, catalyzed by **2** (a) and **3** (b), showing the regioselective formation of 1,4-NADH (purple) and 1,6-NADH (blue) ([NAD+] = 5 mM, [cat] = 100 μM, buffer phosphite 0.4 M, pH = 6.58, T = 313 K).

Table S2. Kinetic data for NAD^+ hydrogenation with **1-3** (phosphite buffer 0.4 M, pH 6.58, 313 K)

Catalyst	[cat] (μM)	[NAD^+] (mM)	TOF (h^{-1})
	7.5	0.25	1252 ± 125
	7.5	0.5	2949 ± 295
	7.5	1	5749 ± 575
	7.5	2	8986 ± 899
	7.5	4	10780 ± 1078

1	7.5	5	12040 ± 1204
	7.5	6	13090 ± 1309
	7.5	8	11180 ± 1118
	7.5	10	10840 ± 1084
	2.5	5	12190 ± 1219
	5	5	14010 ± 1401
	10	5	13040 ± 1304
1_D	7.5	0.77	2320 ± 232
	7.5	1.25	3572 ± 357
	7.5	2	4555 ± 455
	7.5	4	6572 ± 657
	7.5	6	6417 ± 642
	7.5	10	5176 ± 518
2	7.5	0.25	2023 ± 202
	7.5	0.5	1971 ± 197
	7.5	1	1594 ± 159
	7.5	2	1369 ± 137
	7.5	3	1236 ± 124
	7.5	4	930 ± 93
	7.5	6	426 ± 43
	2.5	0.5	1283 ± 128
	5	0.5	1275 ± 128
	10	0.5	884 ± 88
3	7.5	0.25	2379 ± 238
	7.5	0.5	2928 ± 293
	7.5	1	2290 ± 229
	7.5	2	1703 ± 170
	7.5	3	1207 ± 121
	7.5	4	796 ± 80
	7.5	6	631 ± 63
	2.5	1	2579 ± 258

	5	1	2733 ± 273
	10	1	2680 ± 268

Table S3. Kinetic data for NAD^+ hydrogenation with **1-3** ([HCOOK] = 0.125 M in phosphate buffer 0.1 M, pH 7, 313 K)

Catalyst	[cat] (μM)	[NAD^+] (mM)	TOF (h^{-1})
	7.5	0.77	458 ± 46
	7.5	1.25	425 ± 43
1	7.5	2	381 ± 38
	7.5	4	338 ± 34
	7.5	5	226 ± 23
	7.5	6	172 ± 17
	7.5	0.77	445 ± 44
	7.5	1.25	421 ± 42
1_D	7.5	2	388 ± 39
	7.5	4	279 ± 28
	7.5	6	224 ± 22
	7.5	10	135 ± 14
	7.5	0.25	1058 ± 106
	7.5	0.5	941 ± 94
	7.5	1	759 ± 76
2	7.5	2	456 ± 46
	7.5	4	269 ± 27
	7.5	6	208 ± 21
	7.5	0.25	895 ± 90
	7.5	0.5	914 ± 91
	7.5	0.77	910 ± 91

3	7.5	1.25	645 ± 65
	7.5	2	575 ± 56
	7.5	4	449 ± 45

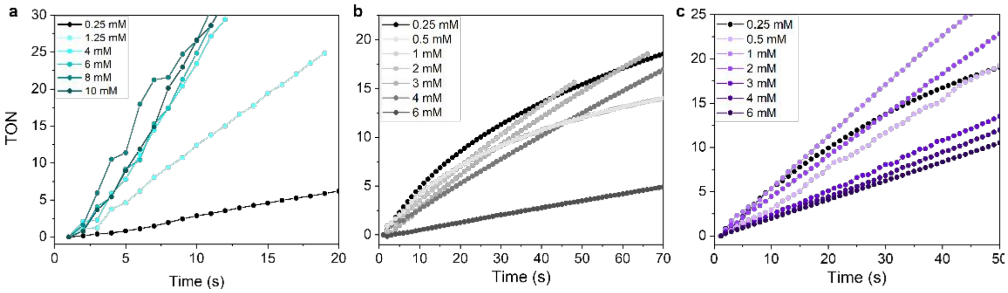

Figure S26. Trends of TON versus t obtained by means of UV-Vis spectroscopy for catalysts **1** (a), **2** (b) and **3** (c) at various [NAD^+] and [cat] = 7.5 μM, phosphite buffer 0.4 M pH 6.58.

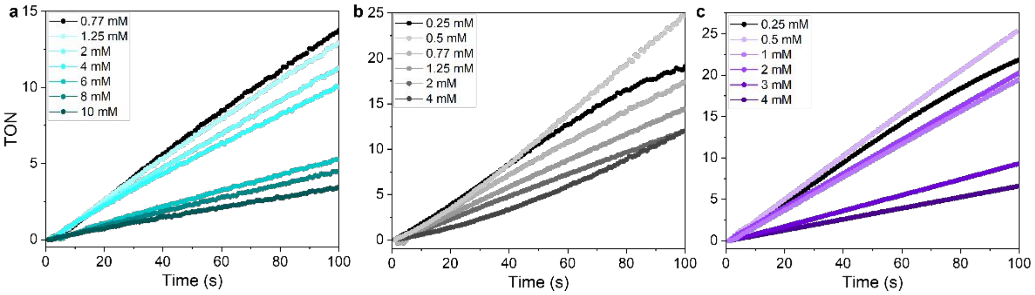

Figure S27. Trends of TON versus t obtained by means of UV-Vis spectroscopy for catalysts **1** (a), **2** (b) and **3** (c) at various [NAD^+] and [cat] = 7.5 μM, [HCOOK] = 0.125 M, phosphate buffer 0.2 M pH 7.

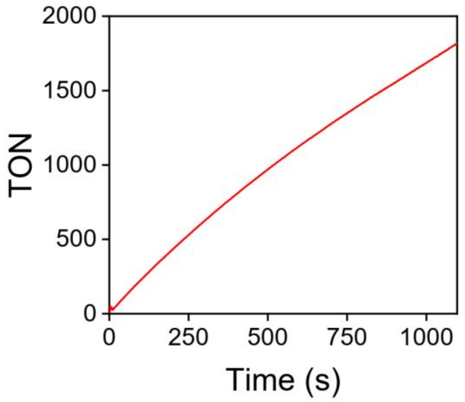

Figure S28. Trend of TON versus t obtained by means of UV-Vis spectroscopy for catalysts **1**. Conditions: [NAD$^+$] = 2.0 mM, [**1**] = 90 nM, 313 K, phosphite buffer 0.4 M, pH 6.58.

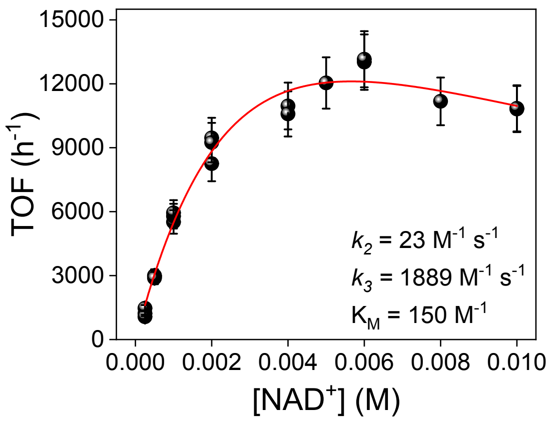

Figure S29. Fitting of kinetic data of catalyst **1** with $H_2PO_3^-$ as a function of NAD$^+$ concentration.
